# Supplementary material for: Efficacy and safety of Cheonwangbosim-dan (Tian Wang Bu Xin Dan) for treatment of mild cognitive impairment: A randomized placebo-controlled pilot trial
Source: PLoS One. 2025 Jul 11;20(7):e0326227. doi: 10.1371/journal.pone.0326227 (PMC12250166; doi:10.1371/journal.pone.0326227)
Supplement: S3 File — (PDF) [file pone.0326227.s003.pdf]

A 24-week randomized, double-blind, placebo-controlled, single-center investigator initiated trial to evaluate the efficacy and safety of Cheonwangbosim-dan on mild cognitive impairment

Introduction

Mild cognitive impairment (MCI) is a transitional state between normal cognition and early Alzheimer's disease (AD). Elderly people with MCI a) have self- or informant-reported cognitive decline, b) have preserved daily living, c) have impairment in one or more of the six cognitive domains, d) do not meet the criteria for AD, and e) do not experience MCI in the context of depression or delirium (1-3). MCI is clinically important since patients with MCI are at high risk of developing AD (4,5). In elderly people aged ≥65 years, 46% of the elderly with MCI develop AD, whereas 3% of healthy elderly develop AD within 3 years (6). Not all the cases of MCI are progressive and precursors to AD. Fortunately, many patients revert to the normal range of cognition (7). Thus, early detection and management of MCI is important for preventing or delaying the progress of MCI to AD (8,9).

Currently, no pharmacologic treatments for MCI have been approved; however, lifestyle modifications or nonpharmacological therapies, such as cognitive stimulation and regular exercise, could be effective in reducing the rate of progression of MCI to AD (7,8,10,11). Nine of the 13 guidance documents (nine consensus statements and four guidelines) cover the management and treatment of MCI, the recommendations for which are classified into four categories: non-pharmacologic interventions, pharmacologic interventions, intervention for risk reduction, and counseling. Three guidelines have not recommended pharmacological intervention. Non-pharmacologic interventions, including acupuncture, physical activity, and nutritional, dietary, and cognitive interventions, have been recommended in seven guidance

documents (12).

Many randomized controlled trials (RCTs) (13-15) and systematic reviews (16-18) on Chinese herbal medicines (CHMs) for MCI treatment have been conducted. Conventional pharmacological treatments for MCI typically act on a single pharmacological target rather than multiple pathologies of MCI (19). However, CHMs, including Cheonwangbosimdan (CWBSD), contain various effective ingredients and have several beneficial effects on multiple targets and pathways, thereby improving cognition. A lot of studies have demonstrated that CHMs are capable of improving cognitive function by protecting against hippocampal neuronal apoptosis, reducing nerve fiber tangling and Aβ deposition, improving hippocampal neuronal mitochondrial function and brain microcirculation, adjusting central cholinergic abnormalities, and protecting cerebral blood vessels (20).

The herbal remedy, known as Cheonwangbosimdan (CWBSD) in Korea, Tian Wang Bu Xin Dan in China, and Tennohosintan in Japan, is often used to treat palpitation, anxiety, and insomnia by stabilizing the patients' minds and providing energy (21-23). CWBSD comprises 15 medicinal herbs: Rehmanniae Radix, Coptidis Rhizoma, Angelicae Gigantis Radix, Asparagi Tuber, Liriopis seu Ophiopogonis Tuber, Zizyphi Semen, Thujae Semen, Schisandrae Fructus, Ginseng Radix, Poria Sclerotium, Platycodonis Radix, Acori Graminei Rhizoma, Polygalae Radix, Salviae Miltiorrhizae Radix, and Scrophulariae Radix. In recent experimental animal studies, CWBSD has been shown to have neuroprotective (24) and vasorelaxant and hypotensive (25) effects and effects against AD (26,27).

Although CWBSD demonstrated significant effects on AD in experimental animal studies, to the best of our knowledge, no RCT has been performed to investigate its efficacy and safety in MCI. Thus, we designed a parallel-arm, double-blinded, randomized, controlled clinical trial to investigate the safety and efficacy of CWBSD for the management of MCI. The findings of our trial would provide clinical trial data concerning the usefulness, safety,

and efficacy of CWBSD in the management of MCI. We present our protocol in accordance with the Standard Protocol Items: Recommendations for Interventional Trials (SPIRIT) reporting checklist (28).

Methods/Design

Aims

We intend to investigate the cognitive improvement effects of CWBSD in patients with MCI.

Hypothesis

We hypothesized that CWBSD will show significant cognitive improvement effects in patients with MCI.

Study design and setting

Our study was approved by the Ministry of Food and Drug Safety (approval No. 32007) and was registered with the Clinical Research Information Service (Registration No. KCT0006787). Our study complies with the Korean Good Clinical Practice guidelines and the principles of the Declaration of Helsinki.

This clinical trial will be a single-center, double-blind, parallel-arm, prospective, randomized controlled trial. Forty-eight eligible participants will be randomly allocated evenly to the CWBSD or placebo group. Dementia and MCI share similar, modifiable risk factors, including heart and cerebrovascular disease, hyperlipidemia, diabetes, hypertension, independent living, rural residence, smoking, and lower education. The management of these risk factors is crucial for preventing MCI progression (9). In patients with MCI, exercise is likely to improve the cognitive function (11); hence, all the participants will be educated on self-management of modifiable risk factors and exercise to prevent MCI progression at

baseline and will receive the trial medication (CWBSD group, CWBSD; placebo group, placebo) once daily for 24 weeks.

Primary outcome will include the changes in the Montreal Cognitive Assessment scale (MoCA-K) scores at 24 weeks following the first intervention. The secondary outcomes will include the changes in the MoCA-K scores at 12 weeks following the first intervention and changes in the scores of the Alzheimer's Disease Assessment Scale-cognitive subscale-3 (ADAS-K-cog 3), Korean Instrumental Activities of Daily Living (K-IADL), Korean Activities of Daily Living (K-ADL), the European Quality of Life Five Dimension Five Level scale (EQ-5D-5L), and Geriatric Depression Scale (GDS) at 12 weeks and at 24 weeks after the first intervention. The trial design is presented in Table 1 and Figure 1.

Table 1. Standard protocol items: recommendations for interventional trials statement (Spirit). The Table shows the enrollment, interventions, and data collection

	**Study period**						
	**Enrollment**	**Allocation**	**Post-allocation**				**Close-out**
**Timepoint**	screening		Visit 1	Visit 2	Visit 3	Visit 4	Visit 5
	week		0	6	12	18	24
**ENROLLMENT**							
Informed consent	X						
Sociodemographic profile	X						
Medical history	X						
Vital Signs	X	X	X	X	X	X	X
Inclusion/exclusion criteria	X						
Allocation		X					

Global Deterioration scale	X					
Korean Mini-Mental State Examination	X					
Korean Version of the Montreal Cognitive Assessment	X					
Geriatric Depression Scale	X					
**INTERVENTIONS**						
Trial medication (CWBSD or placebo)						
Education on self management and exercise		X	X	X	X	
**ASSESSMENTS**						
Change of medical history		X	X	X	X	X
Safety assessment (incidence of AEs)		X	X	X	X	X
Clinical laboratory test	X			X		X
Korean version of the Montreal Cognitive Assessment scale		X		X		X
Korean version of Alzheimer’s Disease Assessment Scale-cognitive subscale-3		X		X		X
Geriatric Depression Scale		X		X		X
Korean Activities of Daily Living (K-ADL), Korean Instrumental Activities of Daily Living (K-IADL)		X		X		X
European Quality of Life five Dimension five Level scale		X		X		X

Figure 1. Study design flow chart

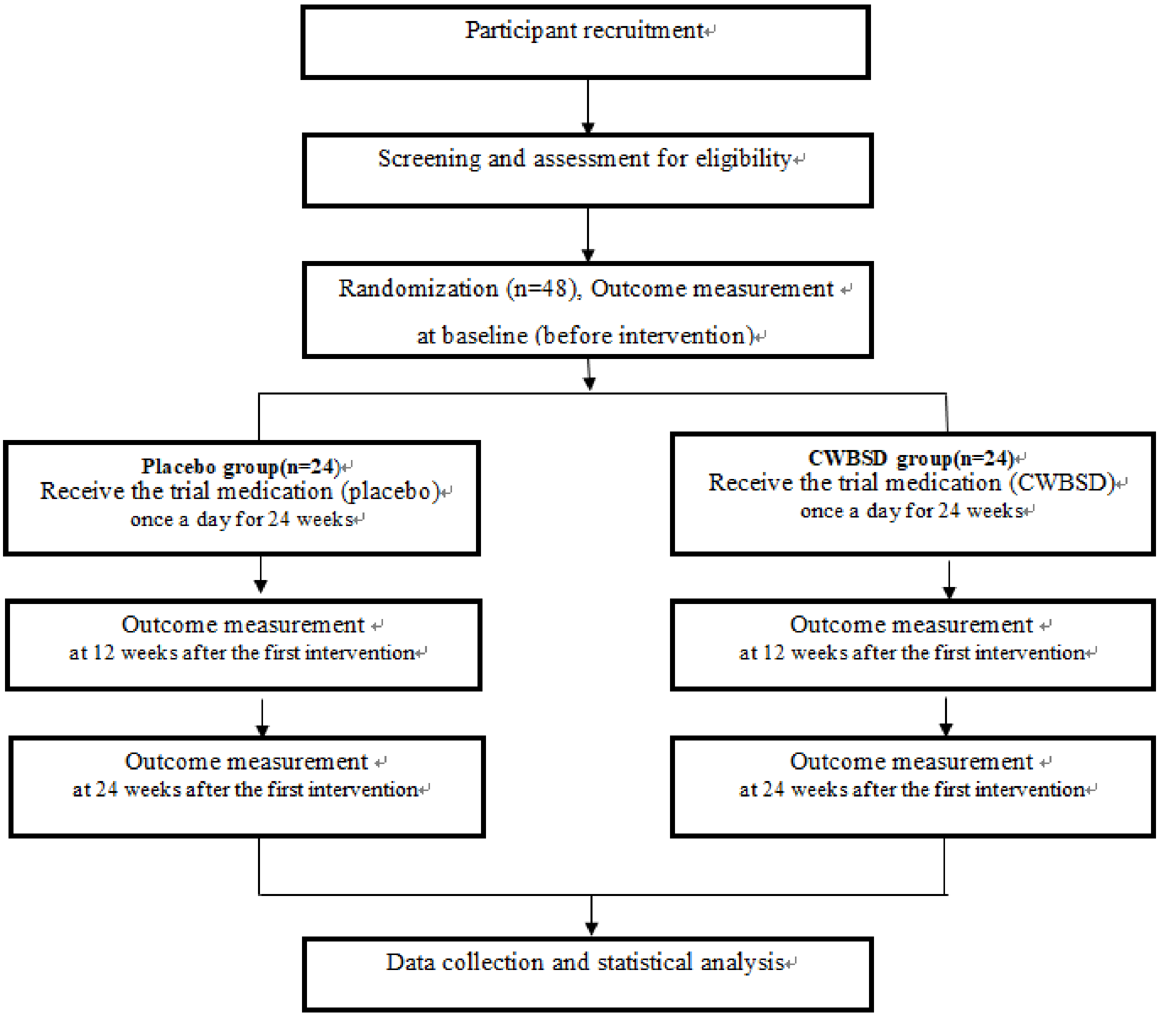

Recruitment

We will recruit participants from the Dongshin University Gwangju Korean medicine Hospital in Republic of Korea via the internet, posters, and local newspapers in hospitals and communities. Interested individuals will receive an explanation of this trial from the clinical research coordinator (CRC) upon their visit at the clinical research center at the hospital and will voluntarily provide written informed consent before participation. All the recruited individuals will be screened using the Global Deterioration Scale, GDS, MoCA-K, and the

Mini-Mental State Examination (K-MMSE).

Whenever the participants visit, the CRC will explain the next visit schedule and will remind the visit schedule by phone call a day prior to the visit. The CRC will adjust the evaluation and visit schedules for each participant to facilitate participation.

Inclusion criteria

1) Adults aged between 55 and 85 years, 2) memory disorder for at least three months preceding screening with fulfillment of the diagnostic criteria for MCI (1-3) (i.e., self- or informant-reported cognitive decline, preserved daily living, impairment in one or more of the six cognitive domains, do not meet the criteria for AD, and do not experience MCI in the context of depression or delirium), 3) MoCA-K scale score of 0–22, 4) K-MMSE score of 20–23, 5) Global Deterioration scale score of 2 or 3, 6) GDS score of 0-18, 7) adequate Korean language proficiency for reliable outcome measurements, and 8) voluntary consent for participation.

Exclusion criteria

1) Diagnosis of AD according to the NINCDS-ADRDA criteria or diagnosis of vascular dementia according to the NINDS-AIREN criteria; 2) a history of structural brain diseases that could cause cognitive decline, such as congenital mental retardation, intracranial space-occupying lesions, stroke, or traumatic brain injury, 3) a history of brain lesions confirmed on brain computed tomography or magnetic resonance imaging within 12 months, 4) presence of a serious disease (e.g., multiple sclerosis, liver, kidney, central nervous system and cardiovascular diseases, Parkinson's and Huntington disease, and cancer), 5) history of treatment for mental illness (depression, serious anxiety, or schizophrenia) or drug/alcohol dependency in the 6 months preceding screening, 6) current management for MCI, such as

cognitive training, medication, or Korean medicine treatment within 4 weeks before screening, 7) difficulties in undergoing outcome measurement attributed to hearing and visual impairments, 8) impaired hepatic function (alanine aminotransferase or aspartate aminotransferase level at least twice the normal upper limit), 9) impaired renal function (creatinine at least twice the normal upper limit), 10) presence of hypertension (170/100 mmHg or greater) or diabetes (over 180 mg/dl of fasting blood glucose), 11) a history of gastrointestinal diseases, anorexia, nausea, and vomiting that could affect the absorption of the CWBSD or placebo, 12) pregnancy or breastfeeding; 13) a history of hypersensitivity to the composition of the CWBSD or placebo, and 14) A person participating in other clinical trials or who has participated in other clinical trials within four weeks of screening for this study.

Dropout and violation criteria

The dropout criteria will be as follows: 1) withdrawal of consent for participating in the clinical trial, 2) incomplete data that can affect the results of the trial, 3) occurrence of a serious adverse event (SAE), or 4) decision to terminate an individual's participation in the trial by the institutional review board (IRB) or principal investigator (PI).

Those who meet the dropout criteria will stop participating in this study.

The violation criteria will be as follows: 1) less than 135 of 168 total doses (<80% compliance with the intervention protocol) and 2) critical error in the protocol or serious deviation in implementation.

Those who meet the dropout and violation criteria will be excluded from the per-protocol set (PPS) analysis.

Ethics

The CRC will explain the purpose and risk of this trial to the participants and their companions. All the participants provided written informed consent before participation.

Randomization and Allocation

After a screening interview by the investigator, the assessor will conduct baseline evaluations for the eligible participants. Forty-eight enrolled participants will be randomly allocated evenly to the CWBSD or placebo groups. The randomization sequence will be generated using the SPSS version 21 software (IBM Corp., USA). The randomization number will be sealed in opaque and sequential envelopes and stored in a double-locked cabinet.

Implementation

The CRC will perform the allocation sequence generation, participant enrolment, group assignment, and participant management.

Blinding

All the investigators, participants, and outcome assessors of this trial will be blinded until the end of study. However, if necessary, such as in the case of the occurrence of SAE, unblinding will be permitted under IRB approval. Randomized labeled and prepacked placebo and CWBSD, which will be identical in taste, smell, and appearance, will be provided by the pharmaceutical company. Randomization and uncovering will be carried out by a statistician with no conflict of interest.

Interventions

The trial medications will be placebo and CWBSD pellets, which will be identical in smell, taste, and appearance. The small, brown pellets will be produced by Hanpoong Pharm &

Food Co., Ltd. (Seoul, Republic of Korea). The detailed composition is shown in Table 2. The production process for the CWBSD is as follows: each component is washed; chopped to a proper small size, and crushed into powder form, followed by mixing of the powders, excipients, and binders. Then, the mixture is converted into pellets, dried, coated, and packed. Both the medications will be administered once daily (total daily dosage, 3 g) for 24 weeks. The participants will visit every 6 weeks and receive a six-week dose of trial medication packed in a sealed box. Each time the participant visits, the CRC will check the medical conditions of the participants and the participant will return their trial medication boxes to evaluate the compliance to improve their adherence to the protocol.

During the study period, the participants will not be allowed to receive other treatment for improving MCI symptoms or use the following drugs: donepezil, rivastigmine, galantamine, memantine, or drugs containing any ingredient of CWBSD. All the participants will receive education on exercise and self-management to improve their cognitive function at each visit. They will be allowed to use pharmacological and non-pharmacological treatment for improving other symptoms that may not affect the results of this study.

Table 2. Composition of CWBSD.

Chinese name	Botanical name	Amount(mg)
Shengdihuang	Rehmanniae Radix	500
Huanglian	Coptidis rhizoma	250
Danggui	Angelicae Gigantis Radix	125
Tianmendong	Asparagi Tuber	125
Wuweizi	Schisandrae Fructus	125

Baiziren	Thujae Semen	125
Suanzaoren	Zizyphi Semen	125
Maimendong	Liriopis seu Ophiopogonis Tuber	125
Renshen	Ginseng Radix	62.5
Jiegeng	Platycodonis Radix	62.5
Yuanzhi	Polygalae Radix	62.5
Xuanshen	Scrophulariae Radix	62.5
danshen	Salviae Miltiorrhizae Radix	62.5
Fuling	Poria sclerotium	62.5

Outcome measures

The primary outcome will include improvements in the cognition evaluated using the MoCA-K at the end of the intervention. The secondary outcomes will include the changes in the MoCA-K scores at 12 weeks following the first intervention and changes in the scores of the ADAS-K-cog 3, K-IADL, K-ADL, EQ-5D-5L, and GDS at 12 and 24 weeks following the first intervention.

The MoCA scale is a brief, validated, and clinician-friendly instrument with high specificity and sensitivity for MCI detection (29).

The ADAS-cog-3 (word recognition, word recall, and orientation) was developed after removing eight tasks demonstrating ceiling effects in MCI from the ADAS-cog-11 to assess only the memory (30).

The GDS was developed as a screening tool to distinguish the symptoms of depression from those of dementia in the elderly (31). The GDS has a simple format that efficiently and

accurately assesses the symptoms of depression, aged from 65 to 85 years. The GDS can be applied to sample cases of younger individuals; however, it may not be the best choice (32).

The K-IADL and K-ADL scales are used to evaluate the physical function of the elderly. The K-IADL scale is used to evaluate complex activities necessary for independent daily life and the K-ADL scale is used to estimate basic activities for daily living (33).

The EQ-5D-5L is a widely used generic patient-reported outcome questionnaire for evaluating the health-related quality of life (34).

Safety assessment

A safety assessment will be carried out by comparing the incidence of adverse events (AEs) and SAEs, changes in the pulse rate, blood pressure, and items of the clinical laboratory test between the two groups.

AEs that could occur in this trial include diarrhea, anorexia, nausea, vomiting, stomach discomfort, itching, skin irritation, and urticaria. All the AEs and SAEs will be recorded in detail, including the degree of severity, potential causal relationships between the AE and trial medication, time of occurrence, and treatment for the improvement of AE by the CRC. They will be reported to the IRB and the IRB will take appropriate action. AEs and SAEs associated with trial medication will be compensated according to the relevant regulations.

Quality control

This study protocol has been developed and reviewed by experts in herbal medicine, MCI, clinical trial, and statistics. Prior to the trial, all the investigators will be trained several times to fully understand the standard operating procedures (SOPs) of this trial and the protocol. An independent clinical research associate will monitor our study and ensure that our clinical trial is carried out in accordance with the SOPs and protocol. The revisions of this protocol

will be reviewed by the IRB of Dongshin University Gwangju Korean medicine Hospital.

Sample size

No adequate preliminary study was conducted upon which the sample size estimation could be based. Thus, a pilot study design was adopted considering the recruitment opportunities, limited funds, and study period. The appropriate sample size for the pilot study is >12 (35). According to a previous study that demonstrated the effects of CHMs on MCI (36), we set the number of groups to 2, effect size to 1.252, two-sided alpha level to 0.05, statistical power to 0.95, and a maximum dropout rate to 25%. Based on these parameters, 48 participants (24 in each group) will be required. Since our trial is a pilot RCT, the sample size will be insufficient to determine the safety and efficacy of CWBSD for MCI. The results of our study would provide clinical trial data concerning the safety, efficacy, and usefulness of CWBSD in patients with MCI.

Statistical analysis

The final data will be analyzed by a statistician with no conflict of interest. The primary analysis population for assessing the efficacy will be a full analysis set (FAS) and a supplementary PPS. The results of the analyses between the FAS and PPS will be compared and reflected in the efficacy evaluation. The last observation carried forward method will be used to obtain the missing values. Interim analyses will not be carried out. All the analyses will be carried out at the 5% (two-sided) significance level using the SPSS version 21 software (IBM Corp., USA).

Baseline variables and characteristics will be compared between the two groups for homogeneity test. Categorical data will be compared using the Fisher's exact test or chi-square test. Continuous data will be compared using the Wilcoxon's rank-sum test or

independent *t* test.

Within each group, changes in the MoCA-K, ADAS-K-cog-3, K-IADL, K-ADL, EQ-5D-5L, and GDS scores at 12 and 24 weeks following the first intervention, relative to the baseline, will be evaluated using a Friedman test or one-way repeated measures analysis of variance (RMANOVA) and Wilcoxon's signed rank test or a paired *t* test. The degrees of change in the MoCA-K, ADAS-K-cog-3, K-IADL, K-ADL, EQ-5D-5L, and GDS scores between the two groups will be compared using the analysis of covariance (ANCOVA) with baseline scores as covariates and independent *t*- test or Wilcoxon rank sum test. Sub-analyses will be carried out in accordance with the participants' age (< 70 and ≥70 years).

The incidence of SAEs and AEs will be compared between two groups using the Fisher's exact or chi-square test. Within each group, changes in the blood pressure, pulse rate, and items of the clinical laboratory test at 24 weeks relative to the baseline will be evaluated using a Wilcoxon signed rank or paired *t*-test. The degree of these changes between two groups will be compared using Wilcoxon rank sum test or independent *t*- test.

Data management and confidentiality

All the documents will not reveal the names and will be labeled and recorded using identification codes. All the identification records will be kept confidential and will not be accessible without IRB approval.

At the end of the study, electronic data recorded by the CRC will be stored on a personal password-protected computer and those who are not authorized by the IRB will not allowed to access the data. The case report forms will be kept in a cabinet until three years after the clinical trial ends. The participants will voluntarily provide written informed consent for the publication of individual details.

Dissemination

The final data will be submitted to the IRB of Dongshin University Gwangju Korean medicine Hospital in Republic of Korea. The results of this study will be published in the peer-reviewed journal.

Abbreviations

AD, Alzheimer's disease; ADAS-K-cog-3, Korean version of Alzheimer's Disease Assessment Scale-cognitive subscale-3; AE, adverse event; CHM, Chinese herbal medicine; CRC, clinical research coordinator; CWBSD, Cheonwangbosimdan; EQ-5D-5L, European Quality of Life Five-Dimension-Five-Level scale; FAS, full analysis set; GDS, Geriatric Depression Scale; IRB, institutional review board; K-ADL, Korean Activities of Daily Living; K-IADL, Korean Instrumental Activities of Daily Living; K-MMSE, Korean version of Mini-Mental State Examination; MCI, mild cognitive impairment; MoCA-K, Korean version of the Montreal Cognitive Assessment; PI, principal investigator; PPS, per-protocol set; RCT, randomized controlled trial; SAE, serious adverse event; SOP, standard operating procedure; SPIRIT, Standard Protocol Items: Recommendations for Interventional Trials.

Footnote

Reporting Checklist

The authors have completed the SPIRIT reporting checklist.

Conflicts of interest

All authors have completed the ICMJE uniform disclosure form. The authors have no conflicts of interest to declare.

References

1. Petersen RC, Smith GE, Waring SC, et al. Aging, memory, and mild cognitive impairment. Int Psychogeriatr 1997;9:65-9.

2. American Psychiatric Association. Diagnostic and statistical manual of mental disorders (DSM-5®). Washington, DC, USA: American Psychiatric Association Publishing; 2013.

3. Tangalos EG, Petersen RC. Mild cognitive impairment in geriatrics. Clin Geriatr Med 2018;34:563-89.

4. Petersen RC. Mild cognitive impairment as a diagnostic entity. J Intern Med 2004;25:183-94.

5. Vega JN, Newhouse PA. Mild cognitive impairment: diagnosis, longitudinal course, and emerging treatments. Curr Psychiatry Rep 2014;16:490.

6. Tschanz JT, Welsh-Bohmer KA, Lyketsos CG, et al. Conversion to dementia from mild cognitive disorder: the Cache County Study. Neurology 2006;67:229-34.

7. Sanford AM. Mild cognitive impairment. Clin Geriatr Med 2017;33:325-37.

8. Anderson ND. State of the science on mild cognitive impairment (MCI). CNS Spectr 2019;24:78-87.

9. Jia L, Du Y, Chu L, et al. Prevalence, risk factors, and management of dementia and mild cognitive impairment in adults aged 60 years or older in China: a cross-sectional study. Lancet Public Health. 2020;5:e661-71.

10. Petersen RC. Mild cognitive impairment. Continuum (Minneap Minn) 2016;22:404-18.

11. Petersen RC, Lopez O, Armstrong MJ, et al. Practice guideline update summary: mild cognitive impairment: report of the Guideline Development, Dissemination, and Implementation Subcommittee of the American Academy of Neurology. Neurology 2018;90:126-35.

12. Chen YX, Liang N, Li XL, et al. Diagnosis and Treatment for Mild Cognitive Impairment: A Systematic Review of Clinical Practice Guidelines and Consensus Statements. Front Neurol. 2021;12:719849.

13. Zhang J, Liu Z, Zhang H, et al. A two-year treatment of amnestic mild cognitive impairment using a compound Chinese medicine: A placebo controlled randomized

trial. Sci Rep 2016;6:28982.

14. Tian J, Shi J, Li T, et al. Efficacy and safety of an herbal therapy in patients with amnestic mild cognitive impairment: A 24-week randomized Phase III trial. Evid Based Complement Alternat Med 2017;2017:4251747.

15. Tian J, Shi J, Wei M, et al. Chinese herbal medicine Qinggongshoutao for the treatment of amnestic mild cognitive impairment: A 52-week randomized controlled trial. Alzheimers Dement (N Y) 2019;5:441-9.

16. Dong L, May BH, Feng M, et al. Chinese herbal medicine for mild cognitive impairment: A systematic review and meta-analysis of cognitive outcomes. Phytother Res 2016;30:1592-604.

17. May BH, Yang AW, Zhang AL, et al. Chinese herbal medicine for mild cognitive impairment and age associated memory impairment: A review of randomised controlled trials. Biogerontology 2009;10:109-23.

18. Dong L, Hyde AJ, Zhang AL, et al. Chinese herbal medicine for mild cognitive impairment using Montreal cognitive assessment: A systematic review. J Altern Complement Med 2019;25:578-92.

19. Steiner GZ, Bensoussan A, Liu J, et al. Study protocol for a randomised, double-blind, placebo-controlled 12-week pilot phase II trial of Sailuotong (SLT) for cognitive function in older adults with mild cognitive impairment. Trials 2018 September 25;19:522.

20. Pei H, Ma L, Cao Y, et al. Traditional Chinese medicine for Alzheimer's disease and other cognitive impairment: a review. Am J Chin Med 2020;48:487-511.

21. Yang XQ, Liu L, Ming SP, et al. Tian Wang Bu Xin Dan for insomnia: A systematic review of efficacy and safety. Evid Based Complement Alternat Med 2019;2019:4260801.

22. Moon SY, Jerng UM, Kwon OJ, et al. Comparative Effectiveness of Cheonwangbosimdan (Tian Wang Bu Xin Dan) Versus Cognitive-Behavioral Therapy for Insomnia in Cancer Patients: A Randomized, Controlled, Open-label, Parallel-Group, Pilot Trial. Integr Cancer Ther 2020;19:1534735420935643.

23. Kim MJ, Bose S, Shin NR, et al. The herbal formula CWBSD improves sleep quality dependent on oral microbial type and tongue diagnostic features in insomnia. J Pers Med 2021;11:325.

24. Kim SH, Kim JW, Kang CH, et al. The effects of Cheonwangbosim-dan and herbs on mouse neuroblastoma 2a cells damaged by hypoxia-reoxygenation. J Orient Neuropsychiatry 2006;17:15-36.

25. Kim B, Jo C, Choi HY, et al. Vasorelaxant and hypotensive effects of

Cheonwangbosimdan in SD and SHR rats. Evid Based Complement Alternat Med 2018;2018:6128604.

26. Choi KW, Jung IC. The effects of Chenwangbosimdan (CBD) hot water extract & ultra-fine powder on the Alzheimer’s disease model. J Orient Neuropsychiatry 2008;19:77-93.

27. Jung IC. Effects of Chenwhangbosim-dan and Sungsimjihwang-tang on protecting microglia and inhibiting acetylcholinesterase and oxidants. Korean J Orient Physiol Pathol 2008;22:120-5.

28. Chan AW, Tetzlaff JM, Altman DG, et al. SPIRIT 2013 statement: defining standard protocol items for clinical trials. Ann Intern Med 2013;158:200-7.

29. Nasreddine ZS, Phillips NA, Bédirian V, et al. The Montreal Cognitive Assessment, MoCA: a brief screening tool for mild cognitive impairment. J Am Geriatr Soc 2005;53:695-9.

30. Kueper JK, Speechley M, Montero-Odasso M. The Alzheimer's Disease Assessment Scale-cognitive subscale (ADAS-cog): modifications and responsiveness in pre-dementia populations. A narrative review. J Alzheimers Dis 2018;63:423-44.

31. Yesavage JA, Brink TL, Rose TL, et al. Development and validation of a geriatric depression screening scale: a preliminary report. J Psychiatr Res 1982-1983;17:37-49.

32. Smarr KL, Keefer AL. Measures of depression and depressive symptoms: Beck Depression Inventory-II (BDI-II), Center for Epidemiologic Studies Depression Scale (CES-D), Geriatric Depression Scale (GDS), Hospital Anxiety and Depression Scale (HADS), and Patient Health Questionnaire-9 (PHQ-9). Arthritis Care Res (Hoboken). 2011;63:S454-66.

33. Won CW, Yang KY, Rho YG, et al. The development of Korean activities of daily living(K-ADL) and Korean Instrumental Activities of Daily Living(K-IADL) scale. J Korean Geriatr Soc 2002;6:107-20.

34. Herdman M, Gudex C, Lloyd A, et al. Development and preliminary testing of the new five-level version of EQ-5D (EQ-5D-5L). Qual Life Res 2011;20:1727-36.

35. Julious SA. Sample size of 12 per group rule of thumb for a pilot study. Pharmaceut Statist 2005;4:287-91.

36. Miao YC, Tian JZ, Shi J, et al. Effects of Chinese medicine for tonifying the kidney and resolving phlegm and blood stasis in treating patients with amnestic mild cognitive impairment: a randomized double-blind and parallel-controlled trial. Zhong Xi Yi Jie He Xue Bao 2012;10:390-7

37. Tian J, Shi J, Zhang X, et al. Herbal therapy: A new pathway for the treatment of Alzheimer's disease. Alzheimers Res Ther 2010;2:30.

38. Lin SK, Yan SH, Lai JN, et al. Patterns of Chinese medicine use in prescriptions for treating Alzheimer's disease in Taiwan. Chin Med 2016;11:12.
