# Supplementary material for: Efficacy and safety of Cheonwangbosim-dan (Tian Wang Bu Xin Dan) for treatment of mild cognitive impairment: A randomized placebo-controlled pilot trial
Source: PLoS One. 2025 Jul 11;20(7):e0326227. doi: 10.1371/journal.pone.0326227 (PMC12250166; doi:10.1371/journal.pone.0326227)
Supplement: S4 File — (DOCX) [file pone.0326227.s004.docx]

**Standard protocol items: recommendations for interventional trials statement.**

|  | **Study period** | | | | | | |
| --- | --- | --- | --- | --- | --- | --- | --- |
|  | **Enrollment** | **Allocation** | **Post-allocation** | | | | **Close-out** |
| **Time point** | screening | | Visit 1 | Visit 2 | Visit 3 | Visit 4 | Visit 5 |
|  | week | | 0 | 6 | 12 | 18 | 24 |
| **ENROLLMENT** |  |  |  |  |  |  |  |
| Informed consent | X |  |  |  |  |  |  |
| Sociodemographic profile | X |  |  |  |  |  |  |
| Medical history | X |  |  |  |  |  |  |
| Vital Signs | X | X | X | X | X | X | X |
| Inclusion/exclusion  criteria | X |  |  |  |  |  |  |
| Allocation |  | X |  |  |  |  |  |
| Korean Mini-Mental State  Examination  Global Deterioration scale  Geriatric Depression Scale  Korean Version of the Montreal Cognitive Assessment | X  X  X  X |  |  |  |  |  |  |
| **INTERVENTIONS** |  |  |  |  |  |  |  |
| CWBSD or placebo prescription |  |  | X X X X | | | | |
| Education on exercise and self-management |  |  | X | X | X | X |  |
| **ASSESSMENTS** |  |  |  |  |  |  |  |
| Change of medical history |  |  | X | X | X | X | X |
| Safety assessment  (occurrence of AEs)  Blood chemistry test | X |  | X | X | X  X | X | X  X |
| Korean version of the Montreal Cognitive Assessment scale |  |  | X |  | X |  | X |
| Geriatric Depression Scale  Korean version of Alzheimer’s Disease Assessment Scale-cognitive subscale-3 |  |  | X  X |  | X  X |  | X  X |
| European Quality of Life five Dimension five Level scale  Korean Instrumental Activities of Daily Living (K-IADL), Korean Activities of Daily Living (K-ADL) |  |  | X  X |  | X  X |  | X  X |
