# Supplementary material for: Efficacy and safety of Cheonwangbosim-dan (Tian Wang Bu Xin Dan) for treatment of mild cognitive impairment: A randomized placebo-controlled pilot trial
Source: PLoS One. 2025 Jul 11;20(7):e0326227. doi: 10.1371/journal.pone.0326227 (PMC12250166; doi:10.1371/journal.pone.0326227)
Supplement: S5 File — (DOCX) [file pone.0326227.s005.docx]

**Components of Cheonwangbosim-dan.**

| Chinese name | Botanical name | Amount(mg) |
| --- | --- | --- |
| Shengdihuang | Rehmanniae Radix | 500 |
| Huanglian | Coptidis rhizoma | 250 |
| Tianmendong | Asparagi Tuber | 125 |
| Danggui | Angelicae Gigantis Radix | 125 |
| Baiziren | Thujae Semen | 125 |
| Wuweizi | Schisandrae Fructus | 125 |
| Maimendong | Liriopis seu Ophiopogonis Tuber | 125 |
| Suanzaoren | Zizyphi Semen | 125 |
| Jiegeng | Platycodonis Radix | 62.5 |
| Renshen | Ginseng Radix | 62.5 |
| Xuanshen | Scrophulariae Radix | 62.5 |
| Yuanzhi | Polygalae Radix | 62.5 |
| Fuling | Poria sclerotium | 62.5 |
| danshen | Salviae Miltiorrhizae Radix | 62.5 |

**Appearacne of Cheonwangbosim-dan.**


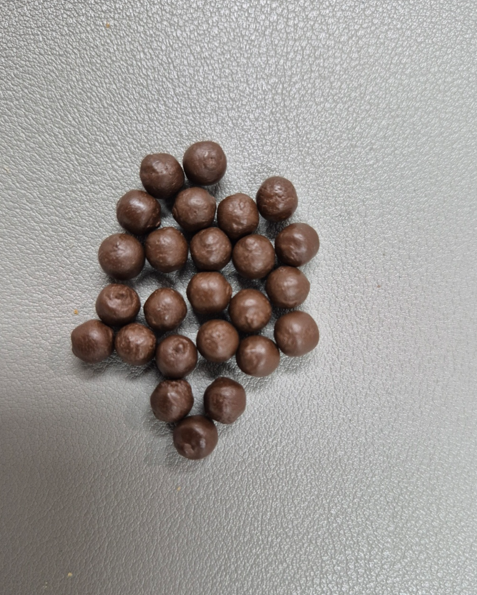


**Packaging of Cheonwangbosim-dan.**

| 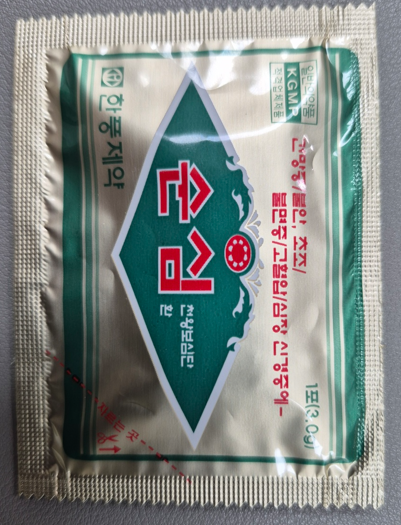 | 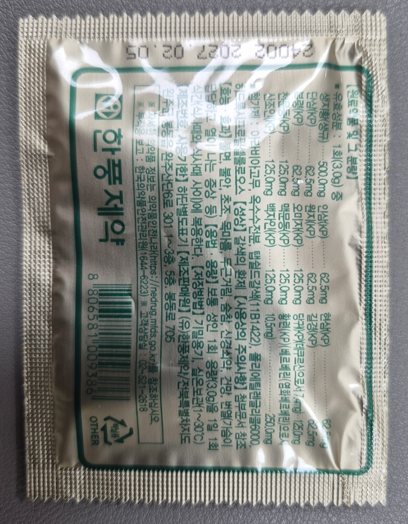 |
| --- | --- |
