# Supplementary material for: Efficacy and safety of Cheonwangbosim-dan (Tian Wang Bu Xin Dan) for treatment of mild cognitive impairment: A randomized placebo-controlled pilot trial
Source: PLoS One. 2025 Jul 11;20(7):e0326227. doi: 10.1371/journal.pone.0326227 (PMC12250166; doi:10.1371/journal.pone.0326227)
Supplement: S6 File — (DOCX) [file pone.0326227.s006.docx]

| Variables | Group(n) | Week 0 | Week 12 | Week 24 | Difference  (W12–W0) | *p* -value | Difference  (W24–W0) | *p* -value | |
| --- | --- | --- | --- | --- | --- | --- | --- | --- | --- |
| Hemoglobin  (g/dl) | CWBSD  (n=24) | 12.5  (11.6-13.2) | 12.5  (12.0-13.1) | 13.0  (12.1-13.7) | 0.15  (0.43) | 0.581 | 0.52  (0.79) | | 0.304 |
|  | Control  (n=24) | 12.4  (11.7-13.0) | 12.6  (11.7-13.1) | 12.7  (12.2-13.3) | 0.10  (0.60) |  | 0.36  (0.39) | |  |
| Hematocrit  (%) | CWBSD  (n=24) | 40.4  (38.0-43.2) | 40.3  (37.4-42.0) | 40.2  (37.2-42.0) | -0.45  (1.33) | 0.679 | -0.68  (3.57) | 0.185 | |
|  | Control  (n=24) | 40.5  (38.0-42.0) | 39.7  (36.9-40.8) | 38.2  (36.9-40.0) | -0.60  (1.70) |  | -1.43  (1.52) |  |  |
| RBC  (10^6^/ml) | CWBSD  (n=24) | 4.3  (3.9-4.5) | 4.2  (3.9-4.5) | 4.2  (3.9-4.5) | 0.00  (0.13) | 0.411 | -0.02  (0.30) | 0.247 | |
|  | Control  (n=24) | 4.2  (3.9-4.4) | 4.2  (3.8-4.4) | 4.1  (3.8-4.4) | -0.03  (0.18) |  | -0.08  (0.15) |  | |
| WBC  (10^3^/µl) | CWBSD  (n=24) | 5.7  (4.9-6.8) | 5.9  (5.2-6.5) | 5.8  (4.8-6.3) | -0.09  (0.65) | 0.447 | -0.16  (1.05) | 0.860 | |
|  | Control  (n=24) | 5.7  (4.8-6.8) | 5.6  (4.5-6.9) | 5.5  (4.5-7.5) | -0.23  (1.03) |  | 0.02  (1.51) |  |  |
| Platelet  (10^3^/µl) | CWBSD  (n=24) | 215.5  (196.5-  245.8) | 202.0  (170.5- 239.5) | 181.0  (157.3- 216.0) | -16.29  (22.19) | 0.967 | -35.58  (30.70) | 0.141 | |
|  | Control  (n=24) | 225.0  (180.5-258.8) | 214.5  (177.3- 233.8) | 170.5  (148.3- 187.8) | -14.83  (31.11) |  | -47.96  (32.52) |  |  |
| AST(SGOT)  (IU/L) | CWBSD  (n=24) | 22.0  (18.0-24.0) | 24.0  (20.3-27.0) | 22.5  (18.0-25.0) | 2.17  (4.65) | 0.146 | -0.08  (4.60) | 0.567 | |
|  | Control  (n=24) | 21.5  (19.0-26.0) | 22.0  (19.3-24.0) | 22.0  (19.0-26.0) | 0.08  (3.44) |  | 0.00  (5.20) |  |  |
| ALT(SGPT)  (IU/L) | CWBSD  (n=24) | 17.5  (14.3-21.0) | 19.0  (16.0-25.0) | 20.0  (15.3-27.3) | 1.79  (4.00) | 0.007 | 3.75  (8.27) | 0.217 | |
|  | Control  (n=24) | 18.5  (15.0-24.8) | 18.0  (15.0-24.3) | 20.0  (16.3-29.0) | -2.29  (7.56) |  | 1.17  (10.97) |  |  |
| ALP  (IU/L) | CWBSD  (n=24) | 140.5  (127.8- 154.5) | 130.5  (105.5- 147.5) | 73.0  (57.3- 109.0) | -21.71  (37.90) | 0.568 | -40.04  (42.88) | 0.395 | |
|  | Control  (n=24) | 141.0  (118.5- 192.8) | 120.5  (86.5- 154.8) | 72.0  (62.0-  91.5) | -28.33  (46.55) |  | -43.25  (42.07) |  |  |
| gamma-GTP  (IU/L) | CWBSD  (n=24) | 14.0  (11.0-17.8) | 17.0  (13.0-20.0) | 19.0  (16.3-22.0) | 2.21  (3.66) | 0.564 | 4.88  (4.35) | 0.499 | |
|  | Control  (n=24) | 14.0  (9.3-22.3) | 17.0  (10.0-25.0) | 16.5  (14.0-26.5) | 2.46  (5.48) |  | 4.21  (7.03) |  |  |
| Total protein  (g/dl) | CWBSD  (n=24) | 6.7  (6.4-7.1) | 7.2  (6.9-7.4) | 7.2  (6.9-7.4) | 0.37  (0.45) | 0.827 | 0.42  (0.43) | 0.633 | |
|  | Control  (n=24) | 6.8  (6.6-7.1) | 7.3  (6.8-7.6) | 7.1  (6.8-7.6) | 0.36  (0.50) |  | 0.34  (0.48) |  |  |
| Albumin  (g/dl) | CWBSD  (n=24) | 4.2  (4.0-4.6) | 4.3  (4.0-4.5) | 4.4  (4.1-4.6) | 0.00  (0.30) | 0.427 | 0.07  (0.29) | 0.588 | |
|  | Control  (n=24) | 4.2  (4.0-4.5) | 4.3  (4.1-4.6) | 4.3  (4.1-4.5) | 0.10  (0.42) |  | 0.06  (0.32) |  |  |
| Glucose  (mg/dL) | CWBSD  (n=24) | 101.5  (93.3- 106.8) | 105.5  (99.3- 109.0) | 103.0  (94.5- 111.3) | 6.08  (7.96) | 0.286 | 4.25  (6.12) | 0.449 | |
|  | Control  (n=24) | 95.5  (90.3- 106.5) | 97.5  (92.3- 109.6) | 98.5  (91.0- 107.8) | 3.88  (9.35) |  | -0.76  (19.82) |  |  |
| Total bilirubin  (mg/dL) | CWBSD  (n=24) | 0.8  (0.5-0.9) | 0.8  (0.6-0.9) | 0.8  (0.7-1.0) | 0.06  (0.08) | 0.776 | 0.13  (0.19) | 0.931 | |
|  | Control  (n=24) | 0.7  (0.6-1.1) | 0.7  (0.6-0.9) | 0.8  (0.7-1.2) | 0.06  (0.10) |  | 0.13  (0.19) |  |  |
| BUN  (mg/dL) | CWBSD  (n=24) | 14.6  (12.5-17.6) | 13.3  (10.8-17.3) | 14.9  (12.7-17.3) | -0.72  (3.78) | 0.983 | 0.20  (1.88) | 0.663 | |
|  | Control  (n=24) | 14.0  (11.7-17.6) | 12.8  (11.3-15.7) | 13.7  (12.6-18.0) | -0.82  (2.79) |  | 0.20  (2.84) |  |  |
| Creatinine  (mg/dL) | CWBSD  (n=24) | 0.5  (0.4-0.6) | 0.6  (0.4-0.7) | 0.6  (0.5-0.9) | 0.05  (0.16) | 0.412 | 0.12  (0.17) | 0.427 | |
|  | Control  (n=24) | 0.5  (0.4-0.6) | 0.6  (0.4-0.7) | 0.6  (0.5-0.7) | 0.06  (0.13) |  | 0.12  (0.09) |  |  |
| Total Cholesterol  (mg/dL) | CWBSD  (n=24) | 177.5  (134.5- 207.3) | 196.0  (139.3- 214.3) | 171.0 (134.8- 208.5) | 10.29  (16.98) | 0.568 | 0.63  (18.00) | 0.325 | |
|  | Control  (n=24) | 187.5 (166.5- 209.3) | 198.0  (176.5- 221.8) | 185.5 (170.0- 200.0) | 7.04  (24.35) |  | -7.21  (26.96) |  |  |
| Triglyceride  (mg/dL) | CWBSD  (n=24) | 88.5  (75.5- 111.8) | 93.0  (75.0- 137.0) | 108.5  (83.8- 121.5) | 7.42  (33.65) | 0.820 | 9.79  (27.86) | 0.844 | |
|  | Control  (n=24) | 103.5  (72.5- 127.0) | 112.5  (73.0- 131.0) | 104.5  (78.8- 136.3) | 1.38  (46.84) |  | -0.83  (39.33) |  |  |
| Sodium(Na)  (mEq/L) | CWBSD  (n=24) | 144.0  (143.0- 145.0) | 144.0  (143.0- 146.0) | 143.0  (142.3- 144.8) | 0.67  (1.66) | 0.912 | -0.25  (1.80) | 0.339 | |
|  | Control  (n=24) | 143.0  (143.0- 144.8) | 144.0  (143.0- 144.8) | 143.0  (142.0- 144.0) | 0.04  (1.46) |  | -0.88  (1.92) |  |  |
| Potassium(K)  (mEq/L) | CWBSD  (n=24) | 4.6  (4.1-4.7) | 4.4  (4.1-4.7) | 4.6  (4.2-4.7) | -0.02  (0.39) | 0.470 | 0.06  (0.36) | 0.033 | |
|  | Control  (n=24) | 4.5  (4.2-4.7) | 4.4  (4.2-4.6) | 4.3  (4.1-4.6) | -0.09  (0.30) |  | -0.14  (0.28) |  |  |
| Chloride(Cl)  (mEq/L) | CWBSD  (n=24) | 106.0  (105.0- 107.0) | 106.0  (105.0- 107.8) | 105.0  (104.0- 107.0) | 0.25  (1.67) | 0.329 | -0.33  (1.71) | 0.306 | |
|  | Control  (n=24) | 106.0  (105.0- 108.0) | 105.0  (105.0- 107.0) | 105.0  (103.0- 106.8) | -0.46  (1.38) |  | -1.13  (1.96) |  |  |

**Values are expressed as mean (standard deviation) or medians (Q1-Q3)**

***p* –value for the between-group comparison using a Mann–Whitney U test**
