# Supplementary material for: Efficacy and safety of Cheonwangbosim-dan (Tian Wang Bu Xin Dan) for treatment of mild cognitive impairment: A randomized placebo-controlled pilot trial
Source: PLoS One. 2025 Jul 11;20(7):e0326227. doi: 10.1371/journal.pone.0326227 (PMC12250166; doi:10.1371/journal.pone.0326227)
Supplement: Attachment — Submitted filename: Response to Reviewers.docx [file pone.0326227.s008.docx]

Dear Editor,

We are immensely grateful for the constructive comments and the opportunity to revise our manuscript. Point-by-point responses to the journal requirements and the reviewers’ comments are provided below. The corresponding changes have been highlighted in the revised manuscript with tracked changes.

Additional Editor Comments:

This is a good study.
There is novelty in this topic.
Please give some details and improvement in this part:
- Table 1 and table 2, I do not think is necessary. The authors can give it as supplements.

**Response.** We sincerely appreciate your encouraging and uplifting comments. As per your invaluable suggestion, we have provided tables 1 and 2 as S4 and S5 files, respectively.

- Please consider any co-morbidities and co-medication in baseline characteristic.

**Response.** We appreciate your invaluable suggestion. In a prior study [1], modifiable risk factors for MCI were identified, including smoking, hypertension, diabetes, hyperlipidemia, heart disease, and cerebrovascular diseases. Consequently, we focused on examining the homogeneity of baseline characteristics, encompassing these comorbidities as well as concomitant medication usage. Furthermore, table 1 has been updated to incorporate these factors, namely smoking, hypertension, diabetes, hyperlipidemia, heart disease, and cerebrovascular disease, as dependent variables.

- Please follow the CONSORT guideline.

**Response.** We have attached the CONSORT 2010 checklist as the S2 file in the supporting information.

- Please consider any confounders in co-medication.

**Response.** Thank you for bringing this up. We have performed a homogeneity test to compare the use of medication for hypertension, diabetes, hyperlipidemia, heart disease, and cerebrovascular disease between the two groups, as detailed in table 1. The results indicated no significant differences in medication usage for these conditions at baseline between the two groups. Consequently, we determined that it was unnecessary to perform an ANCOVA with co-medication as a covariate.

- The graph should be used to describe the improvement.

**Response.** In accordance with your recommendation, we have presented the changes in the MoCA-K scores in figure 2. Additionally, Figures 3 and 4 present the results of the sub-analysis stratified by age. We have also illustrated the variations in secondary efficacy outcomes in figure 5, with the age-stratified sub-analysis results further detailed in figures 6, 7.

- The discussion should be begin with the main finding of your study.

**Response.** As per your invaluable recommendation, we have revised the discussion section to begin with the main findings of our study as follows: “ **Discussion**

The main findings of our study reveal that CBD demonstrated an accepable safety profile and exhibited a significant effect in short-term memory functions. However, it failed to show significant improvements in other cognitive domains, quality of life, daily activities, or depression among patients with MCI. (Page 18, lines 348–351)”

- The conclusion should be concise and clear.

**Response.** We appreciate your insightful comment. In accordance with your suggestion, we have revised the conclusion section to read: “Our findings indicate that a 24-week treatment regimen with CBD was safe and significantly enahnced short-term memory in patients with MCI. Further rigorously designed clinical studies are pertinent to validate the efficacy of CBD in the managemnt of MCI.” (Page 20, line 408-410)

**Reference**

1. Jia L, Du Y, Chu L, Zhang Z, Li F, Lyu D, et al. Prevalence, risk factors, and management of dementia and mild cognitive impairment in adults aged 60 years or older in China: A cross-sectional study. Lancet Public Health. 2020;5: e661-e671. doi: [10.1016/S2468-2667(20)30185-7](https://doi.org/10.1016/s2468-2667(20)30185-7).
