# Supplementary material for: Efficacy and safety of Cheonwangbosim-dan (Tian Wang Bu Xin Dan) for treatment of mild cognitive impairment: A randomized placebo-controlled pilot trial
Source: PLoS One. 2025 Jul 11;20(7):e0326227. doi: 10.1371/journal.pone.0326227 (PMC12250166; doi:10.1371/journal.pone.0326227)
Supplement: Attachment — Submitted filename: response to Reviewers_am.docx [file pone.0326227.s009.docx]

We are very grateful for the constructive comments and the opportunity to revise our manuscript.

Point-by-point responses to the reviewers’ comments are provided below. The corresponding changes have been highlighted in the revised manuscript with track changes.

**Question:** Reviewer #1: The manuscript can be further improved based on the comments below.
Line 152, who are the outcome assessors is to be stated.

**Response:** Thank you for your comments. Outcome assessors are those who evaluate the efficacy outcome, including MoCA-K, ADAS-K-cog 3, GDS-KR, EQ-5D-5L, K-ADL, and K-IADL, as well as safety outcomes. They were received training for the assessment tools employed in our study. We have provided a more detailed description of the outcome assessors as follows: “Throughout the trial, all participants, outcome assessors who were received training for the assessment tools employed in our study and evaluated the efficacy and safety outcomes, and the investigators remained blinded to the group assignments.” (Page 7, lines 156–158 )

**Question:** Line 156-158, the sentence to be placed in Line 151.

**Response:** Thank you for your valuable comments. Following your suggestion, we have revised the randomization and blinding subsection as follows: “The randomization numbers were sealed in opaque envelopes and stored in a double-locked cabinet. An independent investigator, who was not involved in the trial, was responsible for generating the allocation sequence, whereas a clinical research coordinator managed the participant enrollment and group assignments.

Throughout the trial, all participants, outcome assessors who were received training for the assessment tools employed in our study and evaluated the efficacy and safety outcomes, and the investigators remained blinded to the group assignments.” (Page 7, line 152-158)

**Question:** Line 205, whether the effect size 1.252, was derived from the intervention group alone or after comparison with the control group is to be clearly stated.
**Response:** Thank you for your valuable comments. The effect size was calculated based on the comparison between the intervention group and the control group. As there were no existing data demonstrating the efficacy of CWBSD using MoCA-K for sample size calculations, we opted for a pilot study design. The sample size was determined based on a previous randomized controlled trial (RCT) investigating the effects of Chinese herbal medicine using MMSE and ADAS-cog in patients with MCI. The effect size (1.252) was obtained from the differences in pre- and post-treatment scores of ADAS-cog between the Chinese herbal medicine and control group in the referenced RCT.

Since access to the aforementioned article was not available, we have revised the sample size estimation subsection as follows: “We could not find any existing data showing the efficacy of CWBSD using MoCA-K, which is required for sample-size calculations. Therefore, we adopted a pilot-scale study design. The appropriate sample size for the pilot study was >12 [37]. As our study was a pilot trial, formal sample size estimation was not conducted. Instead, considering the sample size of a previous pilot study that explored the efficacy and safety of Chinese herbal medicine for MCI [16], 48 participants were recruited and equally distributed, with 24 participants assigned to each group. The sample size may be insufficient to determine the effects of CWBSD on MCI. The findings of our study provide preliminary data on the effects of CWBSD on MCI.” (Page 10, line 222–230)

**Question:** Line 212, the justification to use LOCF is to be mentioned e.g. MCAR etc.

**Response:** Thank you for your valuable comments. Last observation carried forward is a common statistical approach to analysis of longitudinal repeated measures data where some follow-up observations may be missing. Following your suggestion, we have revised the statistical analyses subsection as follows: “The efficacy evaluation was performed using a full analysis set. Because the missing variable was only affected by random factors (missing completely at random), missing values were imputed using the last observation carried forward technique. (Page 11, line 233–236)

**Question:** Line 223, one or two-tailed test for Fisher’s exact test is to be stated.

**Response:** Thank you for your comments. Since we did not employ Fisher’s exact test in our statistical analyses, it was omitted from mention in the statistical analysis section in the methodology.

**Question:** Line 234 & Line 236, PPS or PP?
**Response:** We sincerely regret any confusion. The mention of the per protocol set in our manuscript was an error on our part. In the original protocol, the analysis of treatment efficacy was intended to be conducted on the full analysis set with a supplementary per protocol set. However, we have since revised our statistical analysis methods and obtained IRB approval to exclude the supplementary per protocol set. Consequently, we have removed all content related to the per protocol set from the manuscript.

**Question:** For inventories such as GDS, EQ-5D-5L, whether Korean version was used is to be clearly stated. Information on the validity/reliability of the instruments used in the study should be briefly described.
**Response:** Thank you for your valuable comments. The GDS and EQ-5D-5L utilized in our study are the Korean versions of these questionnaires. We have provided information on the validity and reliability of the instruments used in the study in the efficacy outcome subsection as follows: “The Montreal Cognitive Assessment Scale is a commonly used instrument for screening MCI in older individuals, measuring cognitive functions across various domains. It is a 30-point test evaluating the cognitive domains of executive function, concentration, attention, working memory, short-term memory recall, visuospatial abilities, orientation to place and time, and language [28]. The MoCA-K is a brief, suitable, and reliable tool for screening MCI patients. The optimal cut-off score of the MoCA-K (22/23) is three points lower than the original MoCA, which may be due to low education years and cultural differences [29].

The Geriatric Depression Scale is a widely used self-report screening instrument to assess depressive symptoms in older adults. It was specifically designed to differentiate between the symptoms of depression and dementia in this demographic. The GDS-KR is a valid and reliable questionnaire for screening major and minor depressive disorders in older people [30].

The Korean version of the Alzheimer’s Disease Assessment Scale is a reliable and valid tool for diagnosing AD and evaluating its severity [31]. The ADAS-K-cog 3, comprising orientation, word recall, and word recognition tasks from the ADAS-K-cog 11, is particularly effective for MCI as it does not exhibit ceiling effects. This tool focuses exclusively on memory assessment [32].

The EQ-5D-5L is a widely used patient-reported health utility instrument for evaluating the quality of life. The Korean version of the EQ-5D-5L is a reliable and valid tool for measuring health-related quality of life in the general population of South Korea [33]. The quality weight for the EQ-5D-5L was based on previous studies [34].

K-IADL and K-ADL are valid and reliable instruments for assessing basic and complex daily activities in older people [35,36]. (Page 9–10, line 193–214)

**Question:** Line 259, the point of analysis e.g. week 12 and week 24 is to be stated. The table to be cited.

**Response:** Thank you for your comments. Following your suggestion, we have revised the efficacy evaluation subsection as follows: “We compared the degrees of change in the MoCA-K, GDS-KR, K-ADL, K-IADL, ADAS-K-cog-3, and EQ-5D-5L scores between the two groups (Week 12 vs. Week 0 and Week 24 vs. Week 0) using the independent *t*-test or Mann–Whitney U test. (Tables 2 and 3)” (Page 13, line 280–282)

**Question:** Table 1, the column p or X^2(p) is confusing. p is to be denoted in the table footnote. Suggest to use just p-value. Some of the p-value symbols are incorrectly labeled. Please re-check the statistical analysis. Denote which one is for the chi-square test and which one is for Fisher’s exact test. The decimal point for the percentage figure is to be consistent. If Fisher’s exact test was not utilized (based on Line 248), the statistical test is to be omitted from mention in the statistical analysis section in the methodology.
**Response:** We sincerely regret for causing confusion. Following your suggestion, we have revised Table 1. In Table 1, we have used only p-values, corrected the symbols for p-values, and consistently revised the decimal point for percentage figures. Since we did not utilize Fisher’s exact test in our statistical analyses, it was omitted from mention in the statistical analysis section in the methodology.

**Question:** For Table 1, having said that, based on CONSORT statement all statistical tests for baseline comparison are to be avoided. The choice to adjust the required variables in the analysis from the baseline characteristics could be based on either clinical relevance, known confounders, visual inspection, sensitivity analyses etc.
**Response:** Thank you for your valuable comments. In a prior study [1], the prevalence of MCI was associated with age, sex, years of education, comorbidities such as hypertension, hyperlipidemia, and diabetes, and other factors. In accordance with your suggestion, in Table 1, we compared the demographic characteristics, including age, gender, years of education, smoking status, and comorbidities (specifically, hypertension, diabetes mellitus, dyslipidemia, and heart disease), as well as baseline cognitive function (measured by global deterioration and K-MMSE), and baseline study variables (measured by MoCA-K, GDS-KR, ADAS-K-cog3, K-ADL, K-IADL, and EQ-5D-5L).

**Question:** Table 2, effect size indices could be presented. The table requires cosmetic changes e.g. alignment of the figures. This applies to all other tables.

**Response:** Thank you for your valuable comments. Following your suggestions, we have included the effect size indices in Table 2 and revised all tables in our manuscript to enhance the visibility of the content.

**Question:** Line 281, the data in Table 2 are median and IQR. As such, the word mean (sd) in Line 281 is to be removed.

**Response:** Thank you for your comments. Following the suggestion of reviewer 2 and after consulting with a statistician not involved in this study, as well as reaching a consensus among the researchers, we have decided to remove the contents related to the analyses on the MoCA-K subscale and sub-analyses by age. Consequently, we have removed Tables 3, 4, 6, 7, Figures 3, 4, 6, 7, and the contents of the MoCA-K subscale from Table 2.

We conducted a Shapiro-Wilk test to assess the normality of data distribution. While the difference (W12-W0) demonstrated a normal distribution, the difference (W24–W0) did not. Therefore, we have presented the data as mean (standard deviation) and median (Q1–Q3) and revised it as follows: “Values are expressed as mean (standard deviation) or medians (Q1–Q3)” (Page 13, line 289)

**Question:** ANCOVA is a parametric test with assumptions of normality. Are those skewed data transformed before analysis? If not transformed, how skewed are the data? More information is to be provided in the methodology section.
**Response:** We apologize for the confusion. It is our mistake. We conducted a Shapiro-Wilk test to assess the normality of data distribution. Except for the difference (Week 12 vs. Week 0) in MoCA-K, all efficacy outcomes did not exhibit a normal distribution. Consequently, we have removed the contents related to ANCOVA from the manuscript.

**Question:** Line 288, if nonparametric tests are used, median and IQR are to be displayed. Likewise, with Table 4 Line 295, Table 5 Line 308, Table 6 Line 318, and Table 7 Line 328.

**Response:** Thank you for your valuable comments. Following the suggestion of reviewer 2 and after consulting with a statistician not involved in this study, as well as reaching a consensus among the researchers, we have removed the contents related to the analyses on the MoCA-K subscale and sub-analyses by age. This includes removing Table 3, 4, 6, 7, Figures 3, 4, 6, 7, and the contents of the MoCA-K subscale from Table 2.

We conducted a Shapiro-Wilk test to assess the normality of data distribution. We have revised the footnotes of Tables 2 and 3 as follows: Table 2 “Values are expressed as mean (standard deviation) or medians (Q1-Q3)

^*^ *p* –value for the between-group comparison using independent t-test.

**^#^***p* –value for the between-group comparison using a Mann–Whitney U test” (Page 14, line 289-291)

Table 3 “Values are expressed as medians (Q1, Q3)

**^#^***p* –value for the between-group comparison using a Mann–Whitney U test” (Page 15, line 300–301)

**Question:** Figure 1, revision required for the analysis section. Excluded and per protocol analysis number is to be separated.

**Response:** We extend our apologies for any confusion that may have arisen. The mention of the per protocol set in Figure 1 was our mistake. In the original protocol, the analysis of treatment efficacy was intended to be conducted on the full analysis set with a supplementary per protocol set. We planned to compare the results of analyses between the full analysis set and the per protocol set to confirm whether there were statistically significant differences between the two groups. However, we have since revised our statistical analysis methods with IRB approval to exclude the supplementary per protocol set. Consequently, we have removed all content related to the per protocol set from Figure 1.

**Question:** Per protocol analysis is to be highlighted or denoted where necessary in the table(s) and/or figure(s).

**Response:** We extend our apologies for any confusion that may have arisen. The mention of the per protocol set in our manuscript was our mistake. In the original protocol, the analysis of treatment efficacy was planned to be conducted on the full analysis set with a supplementary per protocol set. The intention was to compare the results of analyses between the full analysis set and the per protocol set to confirm whether there were statistically significant differences between the two groups. However, we have since revised our statistical analysis methods with IRB approval to exclude the supplementary per protocol set. Consequently, we have removed all content related to the per protocol set from the manuscript.

**Question:** For Figure 2-7, n is to be stated for the CBD group and control group.
**Response:** Thank you for your comments. Following the suggestion of reviewer 2 and after consulting with a statistician not involved in this study, as well as reaching a consensus among the researchers, we have removed the contents related to the analyses on the MoCA-K subscale and sub-analyses by age. This includes removing Figures 3, 4, 6, 7, and the contents of the MoCA-K subscale from Figure 2. We have also described the sample size (n) for the CWBSD and control groups in Figures 2 and 3.

**Question:** The statement * indicates a significant difference between the CBD and control groups for Figure 2-7 is to be denoted in the figures.

**Response:** Thank you for your comments. Following the suggestion of reviewer 2 and after consulting with a statistician not involved in this study, as well as reaching a consensus among the researchers, we have removed the contents related to the analyses on the MoCA-K subscale and sub-analyses by age. This includes removing Figures 3, 4, 6, 7, and the contents of the MoCA-K subscale from Figure 2.

We have inserted the statement '*indicates a significant difference between CWBSD and control groups' in Figures 2 and 3.

**Question:** Reference 35, the DOI link inaccessible <https://doi.org/10.3736/jcim20120407>

**Response:** Thank you for your valuable comments. Since we do not have access to the aforementioned article, we have revised the sample size estimation subsection as follows: “We could not find any existing data showing the efficacy of CWBSD using MoCA-K, which is required for sample-size calculations. Therefore, we adopted a pilot-scale study design. The appropriate sample size for the pilot study was >12 [37]. As our study was a pilot trial, formal sample size estimation was not conducted. Instead, considering the sample size of a previous pilot study that explored the efficacy and safety of Chinese herbal medicine for MCI [16], 48 participants were recruited and equally distributed, with 24 participants assigned to each group. The sample size may be insufficient to determine the effects of CWBSD on MCI. The findings of our study provide preliminary data on the effects of CWBSD on MCI.” (Page 10, line 222-230)

**Question:** Please ensure all links provided in the reference list are accessible. The spacing for some references is inconsistent.

**Response:** Thank you for your valuable comments. We have checked all the links provided in the reference list and verified that they are accessible. Additionally, we have revised the references to ensure consistent spacing.

Reviewer #2: Thank you for the opportunity to review this paper. Whilst this is a well-designed and executed study, the analyses do not appear to be valid. There is no statistically significant group x time interaction (or equivalent) on the primary outcome – the global cognitive screener, MoCA-K. This is OK and should be reported as a null result. However, MoCA-K is divided into sub-tests and these are all analysed and reported without controlling for type 1 error. The relatively small sample is then further stratified into age <70 and >70, which the study was not powered to do and there is no strong argument for the protocol deviation. I have left specific comments section-by-section below.

**Question:** Introduction:
MCI is repeatedly discussed throughout in the context of AD. However, around half of people with MCI do not progress to dementia. Further, only around 45% of people with MCI who progress to dementia develop the AD sub-type. Unless MCI is specifically being characterised as prodromal AD (either with clinical or pathological phenotypes), then this direct assertion to AD should be modified. Instead, MCI should be discussed as a dementia prodrome or dementia risk factor.
**Response:** Thank you for your valuable comments. In accordance with your suggestion, we have described MCI as a dementia prodrome or risk factor for dementia. We have revised the abstract and introduction sections as follows: Abstract “Mild cognitive impairment (MCI), an intermediate condition between healthy cognitive changes due to aging and dementia, is a risk factor for dementia.” (Page 2, line 25–26)

Introduction “Mild cognitive impairment (MCI), regarded as an intermediate condition between healthy cognitive changes due to aging and dementia, is characterized by informant- or self-reported cognitive decline accompanied by preserved daily living [1,2]. It is a significant risk factor for dementia [3,4]. Older adults (≥ 65 years) with MCI have an elevated risk of developing dementia within three years [5]. Many patients with MCI may revert to normal cognition or remain stable, whereas others may exhibit progressive decline, culminating in dementia [6]. Therefore, management and early detection of MCI could aid in the prevention of dementia [7,8].

MCI management aims to hinder, prevent, or even reverse the progression of dementia.” (Page 3, line 50–58)

**Question:** I wonder about the acronym of CBD for the trial IMP. CBD is a commonly used abbreviation for cannabidiol and this may confuse potential readers.

**Response:** Thank you for your valuable comments. Our trial investigational medicinal product (IMP) is Cheonwangbosim-dan pellets. In our manuscript, CBD is an abbreviation for Cheonwangbosim-dan. To avoid confusion, we have revised CBD to CWBSD, the acronym used in other studies.

**Question:** Methods:
Why did the intervention also include education on exercise and self-management? Would this not potentially confound the intervention? What did the education component look like and how was it delivered?

**Response:** Thank you for your valuable comments. Daily life management plays a crucial role in managing the condition in patients with MCI [2], and regular exercise is recommended in practice guidelines for MCI to improve cognitive function [3]. Despite concerns that education on exercise and self-management could impact the results of our study, we ensured consistency by providing all participants with minimal education on self-management and exercises to prevent the progression of MCI while minimizing its impact on the study results.

At each visit, an investigator conducted individual education sessions lasting 10 to 20 minutes using a brochure. The education program covered basic information on MCI, diet, lifestyle management, and exercise guidelines to prevent cognitive function decline. This program was developed based on the dementia guidebook in Korea published by the Ministry of Health and Welfare. We have inserted the contents of the education program into the intervention subsection as follows: “In addition to the pharmacological intervention, all participants received the same education on self-management techniques and physical exercises to prevent the progression of MCI. An investigator conducted an education session with the brochure on each visit for 10 to 20 minutes. The educational program was composed of basic information on MCI, diet, lifestyle management, and exercise guidelines to prevent the deterioration of cognitive function.” (Page 8, line 174–178)

**Question:** Regarding the eligibility criteria. There is no set of diagnostic criteria for MCI in the DSM-V. Instead, it refers to major and mild neurocognitive disorder. Please describe how this was operationalised for the current study.

**Response:** Thank you for your valuable comments. The diagnostic criteria used in our study were based on the relevant criteria outlined in "The Diagnostic and Statistical Manual of Mental Disorders (5th edition)" (DSM-5). Reference 27 refers to the DSM-5. We have provided a detailed description of the diagnostic criteria for MCI as outlined in the DSM-5 in the inclusion criteria subsection as follows: “2) experienced impaired memory persisting for at least three months, meeting the diagnostic criteria for MCI [27], including a) self- or informant-reported cognitive decline, b) preserved daily functioning, c) impairment in one or more of the six cognitive domains, d) absence of dementia criteria, and e) no MCI attributed to delirium or other psychological disorders [e.g., depression];” (Page 5, line 106–111)

**Question:** What is the MCI range for the MoCA-K? The English version uses scores 18 – 25 for MCI, so would 0–22 on the MoCA-K also include dementia?

**Response:** Thank you for your inquiry. The Montreal Cognitive Assessment (MoCA) demonstrates high sensitivity and specificity in distinguishing patients with Mild Cognitive Impairment (MCI) or dementia from cognitively normal older adults. With a cutoff score of 22/23, the MoCA-Korean version (MoCA-K) exhibits excellent sensitivity (89%) and good specificity (84%) for MCI screening. Interestingly, the optimal cutoff point for MoCA-K was observed to be 3 points lower than the original MoCA, potentially attributed to fewer years of formal education [4]. Notably, the study assessing the validity and reliability of MoCA-K did not specify the MCI range; rather, it solely provided the cutoff score for distinguishing MCI or dementia from normal cognition. Consequently, a MoCA-K score of 0-22 encompasses patients with MCI and dementia. In our inclusion criteria, we employed a MoCA-K score of 0-22 to exclude cognitively normal older adults and utilized a Global Deterioration Scale score of 2 or 3 to exclude patients with dementia.

**Question:** Regarding dropout criteria – please provide definition of SAE used or refer to the safety reporting guidelines so that the standardised criteria can be viewed.

**Response:** Thank you for your valuable comments. In accordance with your suggestion, we have inserted the definition of SAE used in our study into the dropout criteria as follows : “1) incidence of any serious adverse event (SAE) (i.e., death or danger to life has occurred, requiring hospitalization, prolonged hospitalization, or permanent or significant disability and impairment);” (Page 7, line 142–145)

**Question:** What exactly is contained within the CBD active? Is this a herbal medicine formulation? If so, which herbs? Are they standardised extracts? More information is needed here.
**Response:** Thank you for your insightful feedback. The CWBSD pellet comprises a brown powdered blend of medicinal herbs including Rehmanniae Radix (Shengdihuang), Coptidis rhizoma (Huanglian), Angelicae Gigantis Radix (Danggui), Asparagi Tuber (Tianmendong), Schisandrae Fructus (Wuweizi), Thujae Semen (Baiziren), Zizyphi Semen (Suanzaoren), Liriopis seu Ophiopogonis Tuber (Maimendong), Ginseng Radix (Renshen), Platycodonis Radix (Jiegeng), Polygalae Radix (Yuanzhi), Scrophulariae Radix (Xuanshen), Salviae Miltiorrhizae Radix (Danshen), and Poria sclerotium (Fuling). Detailed components are provided in the S5 file. The CWBSD was manufactured by Hanpoong Pharm & Food Co., Ltd. (Seoul, Republic of Korea). The manufacturing process involves washing, chopping, and crushing each component into powder form, followed by mixing with excipients and binders. The mixture is then formed into pellets, dried, coated, and packed. The relevant sentence has been incorporated into the intervention subsection accordingly: “The CWBSD pellet, a standardized extract, comprised a brown powdered mixture of medicinal herbs. Detailed components are presented in the S5 file. Placebo and CWBSD pellets, which were identical in smell, taste, and appearance, were produced by Hanpoong Pharm and Food Co. Ltd. (Seoul, Republic of Korea).” (Page 8, line 164–167)

**Question:** What time of day did the once-daily administration of the IMP occur?
**Response:** Thank you for your inquiry. We have incorporated the relevant sentence into the intervention subsection as follows: “CWBSD has been widely used for insomnia [19–21]; therefore, we advised the patients to take CWBSD or placebo once daily after dinner.” (Page 8, line 169–170)

**Question:** For the sample size calculation, what unit of measurement is the expected effect size (1.252) reported in? Cohen’s d?
Which outcome measure was used to determine the sample size calculation?

**Response:** Thank you for your inquiry. Unfortunately, we were unable to locate any existing data regarding the efficacy of CWBSD using MoCA-K, which is necessary for sample size calculations. Therefore, we opted for a pilot study design. Sample size was calculated based on a previous randomized controlled trial (RCT) that examined the effects of Chinese herbal medicine using MMSE and ADAS-cog in patients with mild cognitive impairment (MCI). The effect size of 1.252 was determined from the differences in pre- and post-treatment scores of ADAS-cog between the Chinese herbal medicine and control group. As access to the aforementioned article was not possible, we have revised the sample size estimation subsection accordingly: “We could not find any existing data showing the efficacy of CWBSD using MoCA-K, which is required for sample-size calculations. Therefore, we adopted a pilot-scale study design. The appropriate sample size for the pilot study was >12 [37]. As our study was a pilot trial, formal sample size estimation was not conducted. Instead, considering the sample size of a previous pilot study that explored the efficacy and safety of Chinese herbal medicine for MCI [16], 48 participants were recruited and equally distributed, with 24 participants assigned to each group. The sample size may be insufficient to determine the effects of CWBSD on MCI. The findings of our study provide preliminary data on the effects of CWBSD on MCI.” (Page 10, line 222–230)

**Question:** It’s not clear in the definition of primary and secondary outcome measures that sub-scales from within the MoCA-K will be reported. Was this part of the initial analysis plan, or was it a modification of the protocol? Is it valid to analyse sub-scales from the MoCA-K? The English version is typically reported as the global score (/30) and sub-scales are not analysed due to lack of validity. Re-analysing sub-scales from the MoCA also increases the probability of type 1 error – was testing for multiple comparisons controlled for?
**Response:** Thank you for your valuable feedback. Your input has significantly enhanced the quality of our manuscript. After considering your advice, consulting with a statistician not involved in this study, and reaching a consensus among the researchers, we have decided to remove the content pertaining to the analyses on the MoCA-K subscale. Accordingly, we have revised the Abstract, Results, Discussion, and Conclusion sections as follows:

Abstract : “ We demonstrated that CWBSD treatment was safe and did not significantly improve cognitive function in patients with MCI.” (Page 2, line 42–43)

Result : “The CWBSD group did not exhibit significant improvement in comparison to the control group regarding the MoCA-K total score at Week 24 vs. Week 0 and Week 12 vs. Week 0 (refer to Table 2 and Figure 2).” (Page 13, line 285–287)

Discussion : “The main findings of our study revealed that CWBSD had an acceptable safety profile. However, it failed to show significant improvements in cognitive function, quality of life, daily activities, or depression in patients with MCI. Our clinical trial revealed several findings. First, the CWBSD group did not significantly improve the total MoCA-K score compared with the control group. Second, the two groups had no significant difference regarding the GDS-KR, K-ADL, K-IADL, EQ-5D-5L, and ADAS-K-cog-3 scores. Third, CWBSD did not induce AEs/SAEs or significant negative changes in blood chemistry parameters, blood pressure, or pulse rate after treatment.” (Page 17, line 335–342)

Conclusion : “Our results suggest that a 24-week course of CWBSD treatment is safe. However, it did not significantly improve cognitive function among MCI patients. We anticipate future clinical studies with robust designs and adequate sample sizes to ascertain the efficacy of CBD in MCI management.” (Page 19, line 379–382)

**Question:** The sub-group analysis by age (70) is also likely to be underpowered to detect anything given that this was not accounted for the original sample size calculation. It looks as though each variable has been ‘sliced-up’ multiple times, and now the probability for type 1 error is very high.

**Response:** Thank you for your valuable feedback. Following your advice, consultation with a statistician not involved in this study, and consensus among the researchers, we have opted to remove the content concerning the sub-analyses by age. Consequently, we have revised the Results and Discussion sections as follows:
Result : “ The CWBSD group did not exhibit significant improvement in comparison to the control group regarding the MoCA-K total score at Week 24 vs. Week 0 and Week 12 vs. Week 0 (Table 2 and Figure 2). (Page 13, line 285–287) No secondary efficacy outcome significantly differed in the between-group comparison (Table 3; Figure 3).” (Page 14, line 296–297)

Discussion : “The main findings of our study revealed that CWBSD had an acceptable safety profile. However, it failed to show significant improvements in cognitive function, quality of life, daily activities, or depression in patients with MCI. Our clinical trial revealed several findings. First, the CWBSD group did not significantly improve the total MoCA-K score compared with the control group. Second, the two groups had no significant difference regarding the GDS-KR, K-ADL, K-IADL, EQ-5D-5L, and ADAS-K-cog-3 scores. Third, CWBSD did not induce AEs/SAEs or significant negative changes in blood chemistry parameters, blood pressure, or pulse rate after treatment.” (Page 17 line 335–342)

**Question:** Results:
In text, dropouts are attributed to asked to leave the trial, but in the CONSORT flow diagram (Figure 1), participants withdrew their consent. Please provide more detail here on the reason for consent withdrawal and/or why participants were asked to leave the trial.
**Response:** We sincerely regret any confusion. Due to the withdrawal of consent, participants were requested to discontinue their participation in the trial. To ensure clarity, we have revised the Participants subsection as follows: “Six people in the CWBSD group and four in the control group dropped from the trial throughout the study period due to withdrawal of consent. The withdrawal of consent was not due to AEs but to a simple change of mind.” (Page 11, line 254–257)

**Question:** The figures could be improved by placing together in a single image with multiple panels. There should also be standard error/standard deviation bars.

**Response:** Thank you for your valuable feedback. Following your suggestion, we have included the analysis of the MoCA-K total score as Figure 2, with the MoCA-K subscale contents removed. Additionally, for Figure 3, we have consolidated the figures into multiple panels within a single image. Both figures are presented with standard deviation bars for clarity and consistency.

**Question:** Were variables checked for normality? Did any log-normalisation occur?
**Response:** Thank you for your inquiry. A Shapiro-Wilk test was conducted to assess the normality of the data distribution. Except for the difference (Week 12 vs. Week 0) in MoCA-K, all efficacy outcomes did not exhibit a normal distribution. Mann-Whitney U test was used for between-group comparison excluding the comparison of the difference (Week 12 vs. Week 0) in MoCA-K. Log-normalization was not necessary. We have incorporated the relevant sentence into the statistical analyses subsection as follows: “A Shapiro–Wilk test was performed to check the normality of the data distribution.” (Page 11, line 241)

**Question:** It’s not clear which time points are being compared from the within group comparisons (baseline vs. midpoint, midpoint vs. endpoint, baseline vs. endpoint). I’m also not sure why within-group comparisons are conducted and reported as this was not the primary objective to the trial.

**Response:** Thank you for your feedback. The efficacy outcomes focused on the difference in changes in MoCA-K scores, ADAS-K-cog 3, EQ-5D-5L, GDS-KR, K-ADL, and K-IADL at 12- and 24-weeks post-baseline between the groups. Therefore, conducting within-group comparisons is unnecessary. We have rectified this error by removing the contents related to within-group comparisons from the tables and main text.

**Question:** Compliance data (returned % of unused medication) is not reported. Full details on safety AEs/SAEs should be tabulated.
**Response:** Thank you for your valuable update. No serious adverse events (SAEs) occurred in either group. In both the CWBSD and control groups, only one adverse event (AE) was reported each. Specifically, a participant in the CWBSD group experienced knee pain, while another in the control group reported finger pain. Notably, blood chemistry test parameters revealed a significant increase in potassium levels in the CWBSD group compared to the control group. However, it is important to highlight that these elevated potassium levels remained within the normal ranges. Further details on the safety evaluation are provided in Table 4. Compliance data have been included in the compliance subsection as follows: “Medication compliance was calculated using the following formula:

$\frac{Actualdosage}{mandatorydosage}\times100\left( \text{\%} \right)$

There was no significant difference in medication compliance between the two groups at 24 weeks (Table 5).

(Page 16-17, line 326–329)
**Question:** Other:

elderly is an ageist label. Please see one of the many international guidelines on age-positive language and utilise this throughout (e.g., older people, older adults etc.).

**Response:** Thank you for your comments. In accordance with your suggestions, we have changed ‘elderly’ to ‘older people’ or ‘older adults’.

**Question:** The manuscript would benefit from high-level editing and polishing by a native English speaker.

**Response:** Thank you for your feedback. In response to your comment, we have thoroughly reviewed and edited our manuscript to ensure that the writing is clear, concise, and free from errors in spelling and grammar. This professional editing was carried out by the skilled editors at Editage, a division of Cactus Communications. Upon request, we can provide a certificate of English editing.

**Question:** Tables are difficult to read as the text is split across multiple lines for the same variable (e.g., see ‘Total’ row in Table 2).

**Response:** Thank you for your feedback. In response to your suggestions, we have revised all the tables in our manuscript to ensure that the content is more easily discernible.

**Reference**

1. Jia L, Du Y, Chu L, Zhang Z, Li F, Lyu D, et al. Prevalence, risk factors, and management of dementia and mild cognitive impairment in adults aged 60 years or older in China: A cross-sectional study. Lancet Public Health. 2020;5: e661-e671. doi: [10.1016/S2468-2667(20)30185-7](https://doi.org/10.1016/s2468-2667(20)30185-7).
2. Quinn C, Toms G, Anderson D, Clare L. A Review of Self-Management Interventions for People With Dementia and Mild Cognitive Impairment. J Appl Gerontol. 2016 Nov;35(11):1154-1188. doi: [10.1177/0733464814566852.](https://pubmed.ncbi.nlm.nih.gov/25608870/)
3. Petersen RC, Lopez O, Armstrong MJ, Getchius TSD, Ganguli M, Gloss D, et al Practice guideline update summary: Mild cognitive impairment: Report of the Guideline Development, Dissemination, and Implementation Subcommittee of the American Academy of Neurology. Neurology. 2018 Jan;90(3):126-135. doi: [10.1212/WNL.0000000000004826.](https://www.ncbi.nlm.nih.gov/pmc/articles/PMC5772157/)
4. Lee JY, Dong Woo Lee, Cho SJ, Na DL, Hong Jin Jeon, Kim SK, et al. Brief screening for mild cognitive impairment in elderly outpatient clinic: validation of the Korean version of the Montreal Cognitive Assessment. J Geriatr Psychiatry Neurol. 2008 Jun;21(2):104-110. doi: [10.1177/0891988708316855.](https://pubmed.ncbi.nlm.nih.gov/18474719/)
