# Supplementary material for: Efficacy and safety of Cheonwangbosim-dan (Tian Wang Bu Xin Dan) for treatment of mild cognitive impairment: A randomized placebo-controlled pilot trial
Source: PLoS One. 2025 Jul 11;20(7):e0326227. doi: 10.1371/journal.pone.0326227 (PMC12250166; doi:10.1371/journal.pone.0326227)
Supplement: Attachment — Submitted filename: response_to_Reviewers-resub.docx [file pone.0326227.s010.docx]

We are very grateful for the constructive comments and the opportunity to revise our manuscript.

Point-by-point responses to the reviewers’ comments are provided below. The corresponding changes have been highlighted in the revised manuscript with track changes.

Reviewer #1

**Comment:** The authors have addressed the comments.
Minor comment
The symbol * and ǂ from the ‘p value’ in Table 3 are to be omitted.

**Response:** Thank you for your valuable comments. After considering your suggestion, we have omitted the symbol * and ǂ from the ‘p value’ in Table 3.

Reviewer #3:

In the present study, Kim et.al have investigated the safety and efficacy of
Cheonwangbosim-dan (CWBSD) for treating mild cognitive impairment (MCI). They observed that that CWBSD treatment was safe but did not significantly improve cognitive function in patients with MCI. The study was conducted as a double-blind placebo-controlled design which is robust. The study has several limitations. The conclusion is not clear.

**Comment:** Abstract: The dose of CWBSD can be mentioned in the abstract.
**Response:** Thank you for your valuable comment. As per your suggestion, we have included the dose of CWBSD in the abstract to enhance clarity. The revised abstract now states: “The participants were administered either placebo or CWBSD once daily (at a dose of 3 g) for twenty-four weeks.” (Page 2, line 30–31)

**Comment:** Introduction:
MCI relates to a state when the cognitive ability is not as good as it used to be. Although it refers to problems affecting memory, but could involve a change in problem solving, thinking, attention, concentration, language or visual ability. This needs to be clearly explained in the introduction.
**Response:** Thank you for your valuable comments. As per your suggestion, we have revised the introduction to provide a clearer explanation of mild cognitive impairment (MCI). The revised section now states: “Mild cognitive impairment (MCI) is an intermediate stage between normal cognitive aging and dementia. It is characterized by a decline in one or more cognitive domains, including memory, problem-solving, attention, concentration, language, executive functions, or visuospatial abilities, while daily activities remain largely intact [1, 2].” (Page 3, line 50–53)

**Comment:** MCI is a mild state of cognitive impairment. It is not a disease. It may not be correct to refer to individuals with MCI as “patients”.
**Response:** Thank you for your valuable comment. In accordance with your advice, we have revised MCI patients to individuals with MCI consistently across our manuscript.

**Comment:** What is CWBSD? It needs to be briefly explained in the introduction.

**Response:** Thank you for your valuable comment. As per your suggestion, we have included a brief explanation of Cheonwangbosim-dan (CWBSD) in the introduction to clarify its composition and traditional applications. The revised section now states: “Cheonwangbosim-dan (CWBSD), also known as Tennohosintan in Japan and Tian Wang Bu Xin Dan in China, is a traditional herbal medicine composed of 15 medicinal herbs. It has been widely used in Korea for conditions such as insomnia, depressive and anxiety disorders, and heart palpitations. Given its potential effects on neurological health, it has been investigated for its possible role in cognitive function [19–21].” (Page 4, line 69–73)

**Comment:** Methods
• How was 3g dose of CWBSD administered? Was it a capsule/tablet ? divided dose? This needs to be elaborated
**Response:** Thank you for your query. We have elaborated on the administration method of CWBSD in the intervention subsection. The revised section now states: “The CWBSD pellet, a standardized extract, is a brown powdered mixture of medicinal herbs formulated into a solid pellet form. CWBSD (Product Name: Soon Shim Hwan) is an over-the-counter (OTC) drug, packaged in 3g per dose, and is taken orally as a single daily administration. Participants were instructed to swallow the pellet whole with water. Detailed components, appearance, and packaging are presented in the S5 file. (Page 8, line 167–171)

**Comment:** Physical exercise can have a profound impact on mental function. What kind of exercise was included in the study?
**Response:** Thank you for your query. At each visit, participants received 10–20 minutes of individualized education from a researcher using a brochure. The educational program provided fundamental information on MCI, dietary recommendations, lifestyle modifications, and exercise guidelines to promote cognitive health. This program was based on the Korean dementia guidebook published by the Ministry of Health and Welfare. The exercise component of the program emphasized establishing regular exercise habits, such as daily mild stretching and toning exercises and walking for at least 30 minutes, and instructed participants in simple exercises focusing on stretching, balance, coordination, and relaxation to help maintain cognitive function. We have elaborated on the type of exercise included in the study in the intervention subsection. The revised section now states:

“The educational program included recommendations for exercise, diet, and lifestyle management to support cognitive health. The exercise component of the program emphasized establishing regular exercise habits, such as daily mild stretching and toning exercises and walking for at least 30 minutes, and instructed participants in simple exercises focusing on stretching, balance, coordination, and relaxation to help maintain cognitive function. ” (Page 8–9, line 185–189)

**Comment:** It is also well known that food habits affect mental performance. The diet and lifestyle management need to be elaborated.
**Response:** Thank you for your valuable comments. Our education program also included diet and lifestyle management to prevent cognitive decline. Regarding diet, we recommended consuming foods that support cognitive function, such as fruits, fatty fish, whole grains, vegetables, soy products, and legumes, while limiting intake of sweeteners, unhealthy fats, and refined carbohydrates that can impair cognitive function. Lifestyle management involved managing underlying conditions that can contribute to cognitive impairment, such as hypertension, diabetes, and dyslipidemia, as well as stress management, cognitive training, abstaining from smoking and excessive alcohol consumption, and maintaining close social ties with family and others. We revised the intervention subsection accordingly as follows: “ Dietary recommendations emphasized the consumption of nutrient-rich foods beneficial for cognitive function, such as fruits, fatty fish (e.g., salmon, mackerel, sardines), whole grains, vegetables, soy products, and legumes, while advising participants to limit the intake of sweeteners, unhealthy fats, and refined carbohydrates that may negatively affect cognition. Lifestyle management focused on modifiable risk factors, including the management of underlying conditions such as hypertension, diabetes, and dyslipidemia, stress management, cognitive training, smoking cessation, alcohol avoidance, and maintaining social engagement with family and community members. These guidelines were delivered through individual educational sessions (10–20 minutes) during each visit, supplemented by a standardized brochure based on the dementia guidebook published by the Ministry of Health and Welfare in Korea. However, adherence to these recommendations was not actively monitored during the study.” (Page 9, line 189–200)

**Comment:** In a 24-week study, how was compliance ensured? Were the participants contacted at regular intervals to remind them to take the supplements?

**Response:** Thank you for your query. We ensured participant compliance through structured follow-ups and medication monitoring. The median medication compliance in both the CWBSD and control groups was 96.92%. To facilitate adherence, the CRC provided detailed explanations of visit schedules during each visit and reminded participants of upcoming appointments via phone calls one day prior to their visits. Participants visited the clinic every six weeks and received a six-week supply of the trial medication in a sealed box. To further ensure compliance, the CRC conducted at least one phone check between visits to monitor medication intake. Participants were also required to return any unused medication during their second to fifth visits, allowing adherence to be assessed through pill counts. We have revised the intervention subsection as follows: “The clinical research coordinator (CRC) conducted at least one phone check between visits to monitor medication intake, and participants were instructed to return any unused medication during their second to fifth visits. Adherence was assessed through pill counts to ensure compliance.” (Page 8, line 178–181)

**Comment:** How was safety ensured since the participants visited the hospital only after 12 weeks.

**Response:** Thank you for your comments. We ensured participant safety through regular monitoring of adverse events and physiological parameters. Although hospital visits were scheduled every 6 weeks, safety was monitored continuously throughout the study. Participants were instructed to report any adverse events (AEs) or serious adverse events (SAEs) immediately to the clinical research coordinator (CRC) via phone. Additionally, the CRC conducted periodic phone follow-ups between visits to check for any health concerns or unexpected symptoms. After 24 weeks of treatment, we assessed safety by comparing blood chemistry parameters, blood pressure, pulse rate, and the incidence of AEs/SAEs between the two groups. No SAEs or drug-related AEs were reported. No significant drug-induced changes were observed in blood pressure, pulse rate, or blood chemistry parameters, except for a significant increase in potassium levels in the CWBSD group. However, this increase remained within the normal range. Given the study's duration and funding limitations, post-treatment follow-up was not conducted. Future studies are needed to evaluate the long-term safety of CWBSD in MCI patients beyond 24 weeks. We have revised the abstract and conclusion sections as follows:

***Abstract***

“We demonstrated that CWBSD treatment may be safe and did not significantly improve cognitive function, quality of life, daily activities, or depression among individuals with MCI.” (Page 2, line 41–44)

***Conclusion***

“Our results suggest that a 24-week course of CWBSD treatment was well tolerated, with no observed drug-related adverse events or significant drug-induced alterations in blood pressure, pulse rate, or blood chemistry parameters.” (Page 20, line 411–413)

**Comment:** Results
At what stage did the participants drop out of the trial. Only 38 participants completed the study. The numbers used for analysis is very small. How could the data for 48 participants be used for efficacy analysis?
**Response:** Thank you for your query. Six participants in the CWBSD group and four in the control group withdrew from the study during the medication phase after being assigned to their respective groups due to withdrawal of consent. To address missing data, efficacy analysis was performed using a full analysis set (FAS), and missing values were imputed using the last observation carried forward (LOCF) technique. LOCF is a widely used statistical method in longitudinal studies where missing follow-up observations are replaced with the participant’s last recorded value. This approach assumes that missing data are missing completely at random (MCAR) and helps maintain statistical power despite dropouts.

We have revised the statistical analyses and participants subsections as follows: “Efficacy evaluation was performed using a full analysis set. As missing data were assumed to be missing completely at random (MCAR), missing values were imputed using the last observation carried forward (LOCF) technique.” (Page 12, line 263–266)

**“**Six participants in the CWBSD group and four in the control group dropped out during the medication phase because of withdrawal of consent.” (Page 13, line 283–285)

**Comment:** Safety data on biochemistry and hematology should be included at least as a supplementary table

**Response:** Thank you for your valuable suggestion. In accordance with your suggestion, we have inserted safety data on biochemistry and hematology as supplementary file S6.

**Comment:** In 24 weeks only 2 participants reported an adverse event? Were all events captured?

**Response:** Thank you for your query. All adverse events (AEs) and serious adverse events (SAEs) were carefully monitored and documented throughout the study. Participants were explicitly asked about any adverse symptoms at each visit, and all reported AEs were recorded in detail by the clinical research coordinator (CRC), including the time of occurrence, severity, potential causal relationship to the trial medication, and any treatment received. Additionally, participants were encouraged to report any unexpected symptoms between visits via phone contact with the CRC. The CRC reported all recorded AEs and SAEs to the principal investigator and the Institutional Review Board (IRB) for appropriate action. We have revised the safety outcome subsection as follows:

“All AEs and SAEs were actively monitored throughout the study. Participants were systematically asked about any adverse symptoms at each visit, and they were encouraged to report any unexpected symptoms between visits via phone contact with the CRC. Reported AEs were documented in detail, including the time of occurrence, severity, potential causal relationship to the trial medication, and any treatment provided. The CRC reported all recorded AEs and SAEs to the principal investigator and the IRB to ensure appropriate follow-up and action.” (Page 11, line 243–249)

**Comment:** Discussion
There are several limitations in the study. Although age was significantly higher in CWBSD, all the baseline scores of outcome parameters were almost the same. So this could not be a reason for t CWBSD group not exhibiting a significant improvement.

**Response**: Thank you for your valuable feedback. In response to your suggestions, we have removed the content in the discussion section that the participants’ age may have affected the results.

**Comment:** The exercise and diet could have had an effect in both groups. It would have made an impact to correlate the effect of these factors on cognitive improvement. May be use them as covariate for the analysis.

**Response:** Thank you for your feedback. Since education on daily life management and exercise was provided to both groups, but adherence was not systematically tracked, it was not possible to include these factors as covariates in the analysis. However, we acknowledge that diet and exercise could have had an impact on cognitive outcomes in both groups.

In response to your suggestion, we have revised the discussion section as follows: “First, the education on self-management and exercise provided to both groups may have influenced the results. Lifestyle modifications, including diet and physical activity, play a crucial role in managing MCI [38], and regular exercise is recommended in clinical practice guidelines for improving cognitive function in individuals with MCI [11]. However, as adherence to these lifestyle interventions was not actively monitored, it was not feasible to include them as covariates in our analysis. Future studies should consider systematically tracking diet and exercise adherence to assess their independent effects on cognitive outcomes.” (Page 19, line 374–381)

**Comment:** A dose of 3g/day is high. Increasing the dose can have other deleterious effects
**Response:** Thank you for your feedback. We fully understand your concerns regarding the potential adverse effects of increasing the CWBSD dose. The approved therapeutic dose of CWBSD in the Republic of Korea is 3g/day, and its safety profile at this dosage has been established based on traditional usage and regulatory standards. However, increasing the dose beyond this level may introduce unknown risks, as higher doses have not been systematically evaluated in clinical settings.

In response to your suggestions, we have removed the content in the discussion section that a daily dose may have influenced the results.

**Comments :** The conclusion sentence in incomplete

**Response:** Thank you for your comments. In accordance with your advice, we have revised the conclusion section as follows: “Our results suggest that a 24-week course of CWBSD treatment was well tolerated, with no observed drug-related adverse events or significant drug-induced alterations in blood pressure, pulse rate, or blood chemistry parameters. However, CWBSD did not demonstrate a significant effect on improving cognitive function, quality of life, daily activities, or depression in individuals with MCI. Further studies with longer treatment durations and larger sample sizes are needed to fully assess the long-term safety and efficacy of CWBSD in MCI management.” (Page 20, line 410–416)

**Comment:** Reviewer #4: Due to the small sample size of this study, there are limitations in evaluating efficacy. Therefore, the safety evaluation is of particular importance. In this trial, only two adverse events (AEs) were reported, which appears to be a lower frequency compared to other clinical trials. Could you explain why the number of AEs was so low? Specifically, please provide justification for how AEs were adequately captured in this trial. Additionally, please provide a statistical description of the elevated potassium concentrations in the CWBSD group mentioned in the text, including standard deviation and interquartile range.

**Response:** Thank you for your comments. We understand the importance of ensuring that adverse events (AEs) were adequately captured in this trial. Although CWBSD is an over-the-counter (OTC) herbal medicine that has been widely used for conditions such as insomnia, depressive and anxiety disorders, and heart palpitations in Korea, this does not preclude the need for rigorous AE monitoring in clinical trials. To ensure comprehensive AE reporting, participants were systematically asked about any new or worsening symptoms at each visit, and they were encouraged to report any unexpected symptoms between visits via phone contact with the clinical research coordinator (CRC). All reported AEs and serious adverse events (SAEs) were documented in detail, including time of occurrence, severity, potential causal relationship to the trial medication, and any treatment received. The CRC reported all recorded AEs and SAEs to the principal investigator and the Institutional Review Board (IRB) to ensure appropriate follow-up. We have revised the safety outcome subsection as follows: “All AEs and SAEs were actively monitored throughout the study. Participants were systematically asked about any adverse symptoms at each visit, and they were encouraged to report any unexpected symptoms between visits via phone contact with the CRC. Reported AEs were documented in detail, including the time of occurrence, severity, potential causal relationship to the trial medication, and any treatment provided. The CRC reported all recorded AEs and SAEs to the principal investigator and the IRB to ensure appropriate follow-up and action.” (Page 11, line 243–249)
Additionally, in accordance with your request, we have included a statistical description of potassium concentration changes in the safety evaluation subsection, including the interquartile range (IQR): “A notable exception was potassium levels, which exhibited a significant increase in the CWBSD group (Week 0, 4.6 [4.1- 4.7]; Week 24, 4.6 [4.2- 4.7]; p=0.033) compared to the control group (Week 0, 4.5 [4.2-4.7]; Week 24, 4.3 [4.1-4.6]). However, it is pertinent to note that the elevated potassium levels remained within normal ranges. Detailed blood chemistry test parameters are presented in the S6 file.” (Page 17, line 346–351)
