# Supplementary material for: Efficacy and safety of Cheonwangbosim-dan (Tian Wang Bu Xin Dan) for treatment of mild cognitive impairment: A randomized placebo-controlled pilot trial
Source: PLoS One. 2025 Jul 11;20(7):e0326227. doi: 10.1371/journal.pone.0326227 (PMC12250166; doi:10.1371/journal.pone.0326227)
Supplement: Attachment — Submitted filename: response_to_Reviewers.docx [file pone.0326227.s011.docx]

We are very grateful for the constructive comments and the opportunity to revise our manuscript.

Point-by-point responses to the reviewers’ comments are provided below. The corresponding changes have been highlighted in the revised manuscript with track changes.

Reviewer #1

**Comment:** Line 265-266: Last Observation Carried Forward (LOCF) technique is not recommended if the data is Missing Completely at Random (MCAR).

**Response:** Thank you for your valuable comments. In clinical trials or some longitudinal studies, it is inevitable that missing values will occur. Several statistical approaches have been applied to the analysis of longitudinal data with missing values. The most commonly used imputation methods include complete case analysis (CCA), mean imputation, last observation carried forward (LOCF), hot deck, regression imputation, K-nearest neighbor, the expectation maximization algorithm, and multiple imputation. CCA assumes missing completely at random(MCAR) and includes only those with observed outcome data in the analysis. When the size of the dataset is large enough, analysis could be considered using complete case analysis method where a subject is completely deleted whenever this subject has missing values at any measurement occasion. This causes loss of potentially valuable information about the incomplete cases and MCAR may be hard to justify. Investigators can impute values for missing outcome data rather than ignoring them. The simplest imputation method is the LOCF method that substitutes every missing value with its corresponding last observed value. The LOCF method is a very common approach for handling missing data especially in dropout missingness under MCAR mechanism. This method assumes that the result would not change after the last observed value. Thus, there is no time effect since the last observed value. This situation could be considered as unrealistic in many settings. Thus, LOCF method tends to underestimate the true variability of the data. Compared to CCA method, LOCF method maintains the sample size. However, LOCF method may include bias when dealing with a longitudinal dataset in addition to the long time point of measurement in each interval [1,2].

These approaches should be selected based on the amount of missingness and the missingness mechanism. Some statistical methods are valid only under certain situations with specified missing rates. In other words, there is no unique best method available for all situations.

Despite concerns about the relatively low power and high bias of the LOCF method, we set up a statistical analysis method in the protocol by referring to the statistical analysis method of the previous clinical trial of herbal medicine for mild cognitive impairment [3,4].

As per your suggestion, we have revised the statistical analyses subsection as follows: “Statistical analysis protocol was developed by referring to previous clinical trials of herbal medicine for MCI [38, 39]. Statistical analyses were revised according to IRB directives during the trial period. Efficacy evaluation was performed using a full analysis set. Missing values were imputed using the last observation carried forward (LOCF) technique.” (Page 12, line 263-266)

**Reference**

1. GAD, Ahmed Mahmoud; ABDELKHALEK, Rania Hassan Mohamed. Imputation methods for longitudinal data: A comparative study. International Journal of Statistical Distributions and Applications. 2017; 3(4): 72.

2. Nakai M, Chen DG, Nishimura K, Miyamoto Y. Comparative study of four methods in missing value imputations under missing completely at random mechanism. Open Journal of Statistics. 2014; 4(1): 27-37.

3. Tian J, Shi J, Li T, Li L, Wang Z, Li X, Lv Z, Zheng Q, Wei M, Wang Y. Efficacy and Safety of an Herbal Therapy in Patients with Amnestic Mild Cognitive Impairment: A 24-Week Randomized Phase III Trial. Evid Based Complement Alternat Med. 2017;2017:4251747. doi: 10.1155/2017/4251747.

4. Shin HY, Yim TB, Heo HM, Jahng GH, Kwon S, Cho SY, Park SU, Jung WS, Moon SK, Ko CN, Park JM. Effects of Kami Guibi-tang in patients with mild cognitive impairment: study protocol for a phase III, randomized, double-blind, and placebo-controlled trial. BMC Complement Med Ther. 2022 Dec 2;22(1):318. doi: 10.1186/s12906-022-03805-9.
